# Modeling Gd^3+^ Complexes for Molecular Dynamics Simulations: Toward a Rational Optimization of MRI Contrast Agents

**DOI:** 10.1021/acs.inorgchem.2c01597

**Published:** 2022-07-18

**Authors:** Alexandre C. Oliveira, Hugo A. L. Filipe, João P. Prates Ramalho, Armindo Salvador, Carlos F. G. C. Geraldes, Maria João Moreno, Luís M. S. Loura

**Affiliations:** † Coimbra Chemistry Center - Institute of Molecular Sciences (CQC-IMS), University of Coimbra, 3004-535 Coimbra, Portugal; ‡ Department of Chemistry, University of Coimbra, 3004-535 Coimbra, Portugal; § CPIRN-IPG-Center of Potential and Innovation of Natural Resources, Polytechnic Institute of Guarda, 6300-559 Guarda, Portugal; ∥ Hercules Laboratory, LAQV, REQUIMTE, Department of Chemistry, School of Science and Technology, University of Évora, 7000-671 Évora, Portugal; ⊥ CNC−Center for Neuroscience and Cell Biology, University of Coimbra, P-3004-517 Coimbra, Portugal; # Institute for Interdisciplinary Research - University of Coimbra, Casa Costa Alemão- Polo II, Rua D. Francisco de Lemos, 3030-789 Coimbra, Portugal; ∇ Department of Life Sciences, University of Coimbra, Calçada Martim de Freitas, 3000-393 Coimbra, Portugal; ◆ CIBIT/ICNAS - Instituto de Ciências Nucleares Aplicadas à Saúde, Pólo das Ciências da Saúde, Azinhaga de Santa Comba, 3000-548 Coimbra, Portugal; ¶ Faculty of Pharmacy, University of Coimbra, 3000-548 Coimbra, Portugal

## Abstract

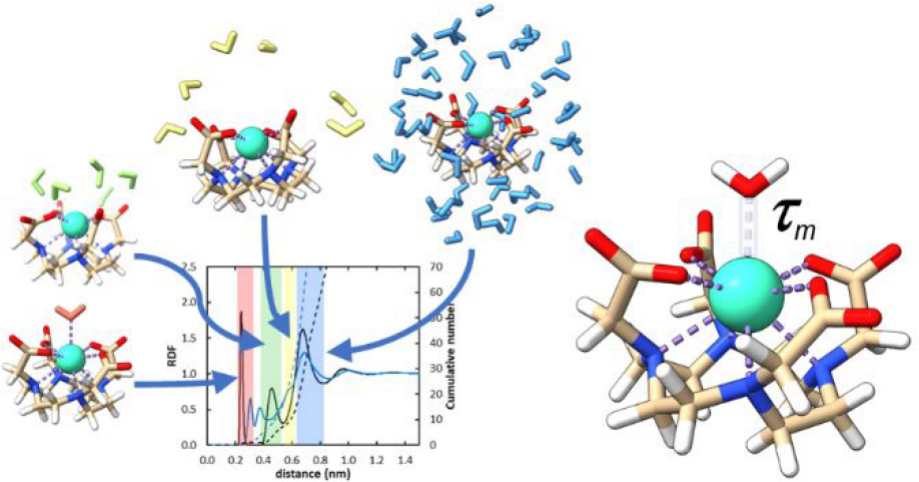

The correct parametrization of lanthanide complexes is of the utmost importance for their characterization using computational tools such as molecular dynamics simulations. This allows the optimization of their properties for a wide range of applications, including medical imaging. Here we present a systematic study to establish the best strategies for the correct parametrization of lanthanide complexes using [Gd(DOTA)]^−^ as a reference, which is used as a contrast agent in MRI. We chose the bonded model to parametrize the lanthanide complexes, which is especially important when considering the study of the complex as a whole (e.g., for the study of the dynamics of its interaction with proteins or membranes). We followed two strategies: a so-called heuristic approach employing strategies already published by other authors and another based on the more recent MCPB.py tool. Adjustment of the Lennard-Jones parameters of the metal was required. The final topologies obtained with both strategies were able to reproduce the experimental ion to oxygen distance, vibrational frequencies, and other structural properties. We report a new strategy to adjust the Lennard-Jones parameters of the metal ion in order to capture dynamic properties such as the residence time of the capping water (τ_m_). For the first time, the correct assessment of the τ_m_ value for Gd-based complexes was possible by recording the dissociative events over up to 10 μs all-atom simulations. The MCPB.py tool allowed the accurate parametrization of [Gd(DOTA)]^−^ in a simpler procedure, and in this case, the dynamics of the water molecules in the outer hydration sphere was also characterized. This sphere was divided into the first hydration layer, an intermediate region, and an outer hydration layer, with a residence time of 18, 10 and 19 ps, respectively, independent of the nonbonded parameters chosen for Gd^3+^. The Lennard-Jones parameters of Gd^3+^ obtained here for [Gd(DOTA)]^−^ may be used with similarly structured gadolinium MRI contrast agents. This allows the use of molecular dynamics simulations to characterize and optimize the contrast agent properties. The characterization of their interaction with membranes and proteins will permit the design of new targeted contrast agents with improved pharmacokinetics.

## Abstract 2

New force-field descriptions are developed and tested for molecular dynamics simulation of MRI contrast agent [Gd(DOTA)]^−^. The best topologies reproduce experimental structural properties and water exchange kinetics. The dynamics of the water molecules in the outer hydration sphere was also characterized.

## 1 Introduction

Medical imaging has a fundamental role in the noninvasive diagnosis of human pathologies. It uses a variety of imaging modalities, namely ultrasound (US), X-ray computed tomography (CT), magnetic resonance imaging (MRI), optical imaging (OI), single-photon emission computed tomography (SPECT), and positron emission tomography (PET).^1^ Some of these techniques use complexes of trivalent lanthanide ions (Ln) as imaging probes, such as the radioisotope ^153^Sm(III) for SPECT^2^ or Yb(III)^3^ and Eu(III)^4^ complexes for OI. MRI has the capacity to noninvasively provide anatomical images with excellent spatial resolution and remarkable inherent contrast, generated by the different intensities of the NMR signals of water protons present in different tissues, due to their different nuclear relaxation rates. Paramagnetic and superparamagnetic probes have been developed as MRI contrast agents (CAs) that accelerate the relaxation process of those protons and further improve the contrast-to-noise ratio of MR images, thus facilitating the diagnosis and shortening the imaging time.^5^

The MRI CAs approved for clinical use are monohydrated, low-molecular-weight Gd(III) complexes capable of shortening the proton longitudinal relaxation times (*T*_1_ = 1/*R*_1_) of neighboring H_2_O molecules, thus increasing their signal intensity in *T*_1_-weighted (*T*_1w_) MR images.^5−8^ Their critical property, representing their efficacy, is the so-called relaxivity (*r*_1_), defined as the ability of the CAs to enhance the relaxation rate (*R*_1_) of water protons per millimolar unit concentration of the metal center. This occurs due to the unpaired electron spins in the 4f orbitals of the Gd^3+^ ion (spin *S*) causing a fluctuating local magnetic field that interacts, through a dipole–dipole mechanism, with the nuclear spins (*I*) of the water protons that come into its close proximity and transmit the paramagnetic effect to the bulk solvent. The overall proton relaxivity is usually divided in inner- and outer-sphere contributions. The former corresponds to the Gd^3+^-coordinated inner-sphere (IS) water molecules, which exchange with bulk water allowing the propagation of the paramagnetic effect to the bulk. The outer-sphere (OS) contribution arises from the paramagnetic relaxation experienced by solvent molecules diffusing in the vicinity of the metal ion. An intermediate sphere is frequently considered, which includes the water molecules transiently interacting with the CA hydrophilic groups. When those water molecules are considered independently, they can be named the first hydration layer and their contribution to water relaxivity is calculated using the formalisms used for the IS.

According to the Swift–Connick and Solomon–Bloembergen–Morgan (SBM) equations, the main parameters controlling the IS contribution are the number of water molecules coordinated in the inner sphere of the paramagnetic center (*q*), their residence lifetime (τ_m_), their Gd^3+^-proton distance (*r*_GdH_), the rotational correlation time of the complex (τ_R_), and the longitudinal (*T*_1e_) and transverse (*T*_2e_) electron spin relaxation times of the metal ion. The OS contribution, describing the effect of the dipolar *I*–*S* intermolecular interactions which fluctuations are governed by the random translational motion of the water molecules, is usually described by Freed’s equation, of which the main parameters are the distance of closest approach of spins *I* and *S* (*d*), the relative diffusion coefficient (*D*) and *T*_ie_ (i = 1,2). The above parameters can be obtained from analyzing experimental data of a combination of proton nuclear magnetic relaxation dispersion (NMRD), temperature-dependent ^17^O NMR relaxation times, and electron paramagnetic resonance (EPR) spectroscopy.^5,6^ The calculation of the contribution of the water molecules in the first hydration layer to the overall relaxivity of the CA is complicated by possible heterogeneity of this water pool.^5,9^ Molecular dynamics simulations (MD), by providing both structural and dynamic properties with atomic resolution, are very useful in the characterization of these CA properties.

**Figure 1:**
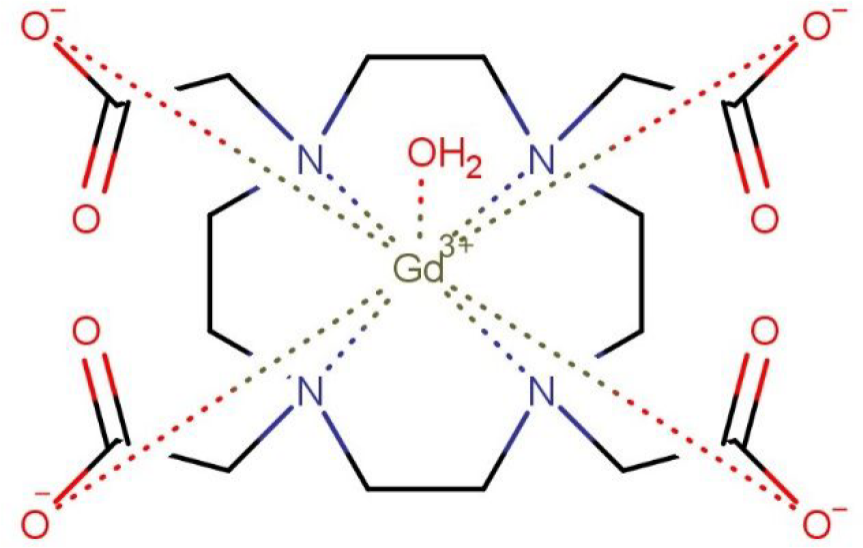
[Gd(DOTA)(H_2_O)]^−^ structure.

One of the MRI CAs most widely used clinically is [Gd(DOTA)(H_2_O)]^−^ (DOTA = 1,4,7,10-tetraazacyclo-dodecane-1,4,7,10-tetraacetic acid) (Figure 1).^10^ This CA is commercially available as DOTAREM, which has been used in neuroimaging for the diagnosis of blood–brain barrier disruption associated with stroke, several types of neurodegenerative diseases and brain tumors.^11^ This contrast agent is thermodynamically and kinetically stable, preventing dissociation of the complex and liberation of toxic Gd^3+^.^5,12^ Ln(DOTA)^−^ complexes occur in solution as a mixture of two distinct isomers with square antiprismatic (SAP) and twisted-square antiprismatic (TSAP) geometries, with characteristic ^1^H and ^13^C NMR spectra for the non-Gd complexes. In the case of Gd^3+^ complexes, the paramagnetic effects of Gd on DOTA nuclear spins prevent the characterization by ^1^H or ^13^C NMR. For those complexes, only the signal from the noncoordinated oxygen atoms may be detected by ^17^O NMR, and it is not possible to distinguish between the SAP and TSAP geometries.^5,13,14^ These isomers constitute two diastereoisomeric pairs of enantiomers, where the macrocyclic ring and the acetate arms have the opposite and the same helicity, respectively.^14^ The SAP conformation is the most stable for [Gd(DOTA)]^−^ in solution, representing around 80% of the population at 298 K.^15^ Recent developments in the contrast agents field are focused on their conjugation with molecular vectors for targeting specific organs and/or pathologies and on increasing their availability in tissues protected by tight endothelia.^5,6^

The wide range of applications of Ln^3+^ complexes has been evidenced by an increase in the number of experimental studies published in this field during the past decades. However, the prediction of the structure and properties of these compounds is done less often, due to the difficulty in the modeling of metal ions by electronic structure or molecular mechanics methods. The most promising methods are those based on density functional theory (DFT),^16^ and their conjugation with molecular dynamics simulations would in theory allow the correct understanding of their dynamic behavior to an extent which is inaccessible by experimental techniques.^17^ The drawback of DFT-based MD simulations is the high computational cost, which limits their applications and becomes prohibitive when complex systems, involving membranes, proteins, and nucleic acids, are studied. For that reason, molecular dynamics with classical mechanics has emerged as a fundamental tool to study biomolecules which are well-described by most common force fields, such as CHARMM,^18^ AMBER,^19^ and GROMOS.^20^ However, the applications of molecular mechanics (MM) to investigate Ln^3+^ complexes are scarce because of the challenge to describe them using classical equations, and indeed most of these common force fields lack parameters for Ln^3+^ ions.^16^ For those reasons, studies of interaction of Ln^3+^ complexes with biomolecules by means of classical mechanics are rare (e.g., refs (21) and (22)).

For modeling metal complexes in MM force fields, several strategies are available: bonded model, nonbonded model, cationic dummy atom approach, and polarizable model approach. In the nonbonded model (also referred as ionic model), the Ln^3+^ ion only interacts with the ligand by means of electrostatic and van der Waals interactions. The bonded model includes covalent bonds between the metal ion and the ligand, namely, stretching, bending, and torsional terms. The cationic dummy atom model is constructed with a variable number of virtual sites according to the coordination number connected to a central metal ion. The dummy sites have fractional charges and masses, which summation with those of the central nucleus gives the formal charge and the total mass of the ion. As far as we know, no cationic dummy atom model has been developed for Ln^3+^ ions. For the polarizable models, there are three basic types of models in use: Drude oscillator (DO), fluctuating charges (FQ) model, and induced dipole model (IDM).^23^

There are some examples of modeling Ln^3+^ ions with the polarizable model using the IDM model with the AMOEBA force field.^24−31^ More sophisticated models take both charge transfer and polarization effects into account, like the work of Marjolin et al. using the SIBFA force field.^32^ However, most of those works are focused on the development of the force field, only studying the free ion in water or in other solvents. A rare exception is the work of Clavaguéra et al., where a Gd(III) polyaminocarboxylate chelate was studied using the AMOEBA force field.^31^

The use of a bonded model can be illustrated by the work of Henriques et al., using the CHARMM22 force field and RESP charges obtained from a single-point calculation by Hartree–Fock (HF) level of theory with 6-31G* basis function for the O, N, C, and H atoms, and for the Gd^3+^ the large-core relativistic effective core potential (LCRECP) of Dolg et al. and the associated (7s6p5d)/[5s4p3d]-GTO valence basis set.^33,34^ The study addressed the molecular isomers that occur in aqueous solution of [Gd(DOTA)]^−^ and [Tm(DOTP)]^5–^. The nonbonded and bonded parameters in the force field were apparently derived empirically in order to reproduce the structural geometries obtained by X-ray crystallography or NMR. The chosen parameters reproduced reasonably well the molecular structures and relative stability of the isomers of [Gd(DOTA)]^−^ and [Tm(DOTP)]^5–^, with root mean square deviation (RMSD) deviations smaller than 0.04 nm.^35^ These authors subsequently tried to optimize the bonded parameters following what they called a heuristic approach, with improved results, further reducing the RMSD.^36^ An attempt was also made on the characterization of the dynamic properties of the water molecules, although only the second sphere could be described due to the small simulation time of only a few nanoseconds. Dimelow et al. used the parameters obtained from Henriques et al.,^35^ converted for use with the AMBER99 force field and using Merz–Kollman charges (MK).^37^ The aim of that work was to study the water exchange mechanism from the metal center to the bulk, using transition path sampling via a Monte Carlo procedure, and to evaluate τ_m_ using a potential of mean force (PMF) method. However, they obtained an overall water residency time of 2100 ps, which they surprisingly compared to an assumed experimental value of 244 ps, 3 orders of magnitude lower than the actual value reported in literature.^38^ Cosentino et al. parametrized [Gd(DOTA)]^−^ as a reference to study the 1-(2-(*b*-galactopyranosyloxy)-propyl)-4,7,10-tris(carboxymethyl)-1,4,7,10-tetraazacyclo dodecane) Gd(III) complex (abbreviated Gd-EgadMe), using the TRIPOS force field and MK charges fitted from *in vacuo* calculation at HF/6-311G**.^39^ The bonded parameters were obtained based on a strategy they had previously adopted for this kind of systems, where they fitted the MM empirical potential to the potential energy surface (PES) obtained by *ab initio* calculations, in a procedure that includes the energies and their derivatives in the fitting.^40,41^ The van der Waals parameters were obtained by means of several short MD simulations choosing the parameters that were able to reproduce the conformational energies and the ion to oxygen distance (IOD), without addressing the dynamics of the water molecules. A work from Borel et al. addressed the outer sphere of several metal complexes. In their parametrization strategy, these authors used the MK method for the charges. To avoid dissociation of the complex, they imposed bonds both between the ligand and Gd^3+^ and between the capping water and the Gd^3+^, the goal being to study the outer sphere. In the case of the [Gd(DOTA)]^−^, they establish that it can be divided into a hydrophobic part, comprising the macrocycle, where no apparent orientation of the water was observed, and a hydrophilic part, comprising the carboxylates, where around 4.3 water molecules are present in this sphere with a residence time of 27.4 ps, forming hydrogen bonds with the carboxylates and in less extent with the inner-sphere water.^42^

The use of the nonbonded model can be illustrated by the study of Bonnet et al., who used the CHARMM all-atom force field, assigning a charge +3 to the free Gd^3+^ ion, and defining its nonbonded Lennard-Jones (LJ) parameters that are able to reproduce the coordination number (8 coordinating water molecules), IOD of approximately 2.4 Å, and the hydration free energy of Gd^3+^ in solution. Additionally, they used distance restraints to ensure the correct coordination sphere of Gd^3+^ with the ligand in study. The aim of the work was to study a probe with only outer-sphere relaxation that can help in the study of the distinct contributions to the overall water proton relaxivity. They studied GdPA (PA = c(AspArgGluProGlyGluTrpAspProGly)) and concluded the existence of a large second-sphere contribution of 30% at high magnetic field to the overall water proton relaxivity.^43^ Yerly et al. studied the physical–chemical properties of several acyclic and macrocyclic Gd^3+^ chelates, including [Gd(DOTA)]^−^, in order to evaluate the behavior of certain parameters that influence the relaxivity, such as the rotational correlation time.^44,45^ Their parametrization strategy also took into consideration a +3 charge in the Gd^3+^ ion. The LJ parameters for the Gd^3+^ ion were chosen in order to reproduce the experimental coordination structure, and no artificial constraints were introduced on the first coordination sphere. Partial charges of the chelating agent were derived from *ab initio* calculation at RHF level with 6-31G** basis sets using the Mulliken method, based on a previous observation that Mulliken charges reproduced the interaction within the complex better than MK charges.^42^ In those studies, the authors characterize some dynamical properties of the complexes, including conformational flexibility and rotational correlation time. A couple of events of exchange of the inner-sphere water in different studied complexes were also observed. However, those events should not have happened, since the authors only simulated each system for 1 ns and experimental data indicate that the residence lifetime is several orders of magnitude longer.

Migliorati et al. parametrized the Ln^3+^ ion series for the LJ and Buckingham potentials in order to reproduce experimental structural properties obtained by X-ray absorption fine structure (EXAFS).^46^ They were able to reproduce the structural properties of the hydrated ions, obtaining results similar to those of a previous work, where they used explicit polarization in the classical force field.^47^ However, they were not able to address the issue that the same results may be obtained with multiple pairs of σ and ε (the zero energy distance and the potential well depth, respectively). The initial σ and ε were taken from a work where those parameters were adjusted by trial and error, until obtaining the hydration structure of Nd^3+^ and Dy^3+^.^48^ From those parameters, Migliorati et al. arbitrarily chose to fix the ε_LnO_ value, and only calibrate σ_LnO_. To solve the multiple combinations of LJ parameters ε and *R*_min_ = 2^1/6^σ (where *R*_min_/2 represents the van der Waals radius of the ion corresponding to the minimum value in the LJ potential) that give similar hydration free energy (HFE) or IOD, Li et al. proposed the noble gas curve (NGC) to provide a quantitative relationship between *R*_min_/2 and ε, reducing the two-dimensional optimization problem to a one-dimensional one. The NGC curve was fitted to experimentally determined *R*_min_/2 and ε values of the noble gas atoms.^49^ These authors developed a series of nonbonded parameters for different metal ions with different oxidation states +1,^50^ + 2,^49^ +3, and +4,^51^ in order to reproduce HFE, IOD, and coordination numbers employing the 12–6 LJ nonbonded model using different water models (SPC, TIP3P, and TIP4P). They realize that this simple model did not reproduce simultaneously the IOD and HFE for highly charged ions, and for that reason, they parametrized these ions with the 12–6–4 LJ-type model. They have further updated a semiautomated method (Python Based Metal Center Parameter Builder, MCPB.py),^52^ based on a previously developed MCPB program,^53^ to build force fields for metal-complexes (such as organometallic compounds and metalloproteins), employing the bonded model or the nonbonded model and using the van der Waals parameters for the metal ions they had previously optimized^49−51^ or from the UFF force field.^54^ This program supports more than 80 metal ions and various AMBER force fields. The force constants parameters for metal-ion-related bonds and angles can be obtained by several methods, and in this program one can choose between empirical,^55^ Seminario,^56^ and *Z*-matrix methods. The empirical method is only supported for the Zn^2+^ ion. The *Z*-matrix and Seminario methods are based on theoretically calculated Cartesian Hessian matrix and submatrices of the Cartesian Hessian matrix, respectively, both obtained from quantum mechanics (QM) calculations. The dihedral parameters are set up with zero energy barriers.^52^

Because Ln^3+^ ions behave as hard Lewis acid cations according to the definition of Pearson,^57^ the chemical bonds they form with a chelator are largely based on electrostatic interactions, with hard anionic ligands being preferred.^58^ For the simulation of lanthanides, the more accurate method would be the use of DFT-MD. However, the high computational cost makes it impractical. In terms of classical models, Li and Merz suggest that the best strategy for the modeling of lanthanide ions is the use of induced dipole model (polarizable model) or the 12–6–4 nonbonded model.^23^ The polarizable model is probably the one that can describe the system the best, since it takes into consideration the polarization effect, and some IDM examples can also include the charge transfer effects. However, it significantly increases the computational cost, reducing its applicability for more complex systems, and most of the MD simulation packages like AMBER,^59^ CHARMM,^60^ GROMACS,^61^ OpenMM,^62^ and LAMMPS^63^ do not handle all the available polarizable models. For that reason, the polarizable model is excluded here, and we focus on unpolarizable models (comprising the cationic dummy atom, nonbonded, bonded, and coarse-grained models), that all of these software packages can handle, adding the fact that they have been optimized to describe biological components like membranes, proteins, and nucleic acids.^23^ As far as we know, no cationic dummy atom approach and coarse-grained models have been used to model Ln^3+^ ions, and we wanted to follow works already developed in this area. The remaining question is the choice between the nonbonded and bonded models. This was rationalized taking into consideration that in order to use the nonbonded model, it would be necessary to use a +3 charge in the Gd. This is contrary to indications from our *ab initio* and DFT calculations that pointed to the existence of some charge transfer, resulting in a Gd charge lower than +3. Additionally, the bonded model ensures that the Gd^3+^ ion is not labile and does not leave the chelate during a simulation, which is particularly important when the object of study is the whole complex (e.g., in the study of the complex interacting with proteins or membranes), making this a convenient model. For those reasons, in the present work we employed the bonded model using the atom charges given by the QM calculation considering the charge transfer from Gd^3+^ to the surrounding chelator, DOTA^4–^. In this way, charge transfer is implicitly taken into consideration in our parametrization. The very high stability of [Gd(DOTA)]^−^ (both thermodynamically and kinetically; conditional stability constant, log *K*′_Gd(L)_ = 19.0 at pH 7.4,^64^ and half-life at 310 K of *t*_1/2_ = 26.4 h at pH 1, leading to an interpolated value at pH 5.0 of 33 years and an extrapolated value of 44 years at pH 7.4),^65,66^ with high conformational rigidity and tight packaging, further justifies the choice of the bonded model as a valid approximation.

As reviewed above, several parametrization strategies have been proposed during the last years for simulating lanthanide complexes by means of MD simulations, but most of these works follow nonsystematic approaches. Excluding the works where polarized force fields were used, the works employing unpolarized force fields can be divided into two groups, concerning the parametrization strategy used for the metal complexes: the bonded or nonbonded model. It is our understanding that the bonded model presents several advantages. It is a representation of the whole complex, prevents the dissociation of the complex in the free ion and ligand, and allows the simulation of the organometallic complex in a specific stereochemistry. The major difficulty encountered by most authors is the optimization of the van der Waals parameters for the metal ion. In most unpolarized force fields, it is modeled by the LJ potential function. Most of the authors did not address the multidimensionality problem of the LJ parameters, and when they do so they only consider the free ion. From the above, there is a clear necessity to update the methodology and the force fields developed so far for lanthanide complexes and to address the problems encountered by the different authors. In this work, we present a systematic study to determine the best parametrization strategy for Ln complexes, using the bonded model and the [Gd(DOTA)]^−^ as reference. The first approach was based on works already published in the field, namely, the attempts by Henriques,^35,36^ who used the CHARMM22 force field, and Dimelow,^37^ who used the AMBER99 force field. The chosen force field was a more recent version of the CHARMM, the CHARMM general force field (CGenFF) version 4.1. It was rapidly realized that this procedure, referred in these articles as a heuristic strategy, does not always follow a rational approach (as the name suggests) and required a large number of optimization steps. For that reason, the capabilities of the MCPB.py tool^52^ were also explored, using the General Amber force field (GAFF). Because the LJ parameters for the Ln series acquired by Li et al.^51^ were obtained for the free metal ion with a formal charge of +3, and since *ab initio* calculations show charge transfer from the metal ion to the surrounding chelate, the best strategy seemed to consist of using those partial charges, considering the charge transfer effect implicitly, and optimizing the LJ parameters for the chelated Gd^3+^, to reproduce the experimental IOD or τ_m_. Although the simple model 12–6 LJ potential cannot simultaneously reproduce the IOD and HFE as noted by Li et al.,^51^ this simple model ensures low computational cost and is supported by most MD software packages, at variance with the 12–6–4 model (that as far as we know is only supported by the Amber software package). Although this simple model has difficulties to address more than one property simultaneously, the new parametrization with the new LJ parameters for Gd^3+^ was able to reproduce a dynamic property, such as τ_m_. For the first time, the true [Gd(DOTA)]^−^ τ_m_ value was correctly accessed with registration of the exchange events, at a cost of a low IOD error. The relatively small value of τ_m_ allowed the direct assessment of the exchange events, instead of calculating the exchange kinetics from the PMF.^67^ These new optimized LJ parameters can be used for similar Gd^3+^ metal complexes. Since the chemistry involving the Ln series is similar,^15^ we believe that the strategies employed in this study can also be extended to all the metal ions of the Ln series, as long as experimental data is available for tuning the LJ parameters. This work opens the possibility not only of addressing fundamental properties of these complexes as contrast agents, such as τ_m_, but also to have a correct parametrization to study their interactions with proteins, membranes or nucleic acids.

## 2 Methods

### 2.1 Simulation and Modeling

All simulations and analysis were carried out with GROMACS 2019^68^ and adapting the protocols from Lemkul,^69^ while the quantum mechanical calculations were carried out with Gaussian 16 software adapting the input examples from Lumpe et al.^70^ For visualization, Avogadro^71^ and VMD^72^ were used. The bonds, angles and dihedrals indexes were obtained with the Open Babel software.^73^ The RESP charges and the modeling using MCPB.py were carried out using the modules in AMBERtools 19^74^ (with a bug correction explained in the Supporting Information Appendix S.I.3), and the conversion of the topology and coordinate files from AMBER to GROMACS package was done using ACPYPE software.^75^

The simulations for the optimization of the topology were carried out using a cubic water box, with a [Gd(DOTA)]^−^ complex placed at the center of the box and a distance of 2 nm to the closest box boundaries. For the MCPB procedures, the TIP3P water model, was used,^76,77^ with the LJ parameters for the hydrogens set to zero, and for the heuristic procedure, a slightly modified TIP3P water model^78^ (mTIP3P, these are the typical water models used with the GAFF and CGenFF force fields, respectively, see section 2.2 for further detail) was employed. A total of 3779 solvent molecules were added in both procedures. For the united-atom procedure, used with the GROMOS 54A7 force field (see Section 2.2.4), 3784 SPC waters^79^ were added to the system. One Na^+^ ion was added for system neutralization. For the [Gd(DOTA)]^−^ complex τ_m_ optimization, due to the necessity of longer simulation times, the box size was reduced (containing 1706 water molecules), and the distance from the complex to the box boundaries was now 1.5 nm. In this case, the box size was at least larger than the longer cutoff distance used for intermolecular interactions (Coulombic and van der Waals) in order to avoid the minimum-image convention violation confirmed by the gmx mindist module of the GROMACS package.^68^

A steepest descent minimization was carried out, followed by a 100 ps *NVT* simulation and 100 ps *NPT* run, both with an integration step of 1 fs. The production simulation was performed under *NPT* conditions, with an integration step of 2 fs. For all simulations, periodic boundary conditions were employed together with the long-range electrostatic interactions modeled by the particle-mesh Ewald (PME) method.^80^ The complex and the solvent together with the sodium were coupled independently to a temperature bath of 298.15 K. Specific parameters of the simulation for each model used are described in Table 1.

**Table 1:** Parameters for Each Simulation Method Procedure^69,81,82^

	heuristic procedure/CGenFF force field	MCPB procedure/GAFF force field	united-atom procedure/Gromos54A7 force field
water model used	mTIP3P	TIP3P	SPC
*T*_control_	Nosé-Hoover^83,84^	Nosé-Hoover^83,84^	Nosé-Hoover^83,84^
*T*_constant_	tau-*t* = 1.0 ps	tau-*t* = 0.5 ps	tau-*t* = 0.5 ps
*P*_control_	Parrinello-Rahman^85^	Parrinello-Rahman^85^	Parrinello-Rahman^85^
*P*_constant_	tau-*p* = 5.0 ps	tau-*p* = 10.0 ps	tau-*p* = 2.0 ps
electrostatic interaction	1.2 nm; PME	1.4 nm; PME	1.0 nm; PME
vdW cutoff method	force-based switch	hard-truncation	hard-truncation
vdW cutoff range	1.0–1.2 nm	1.4 nm	1.0 nm
dispersion correction	no	energy and pressure	energy and pressure
constraint algorithm	LINCS^86^	LINCS^86^	LINCS^86^
constraints	H-bonds	all-bonds	all-bonds

The simulations for the analysis of bonds, angles, dihedrals, RMSD, and IOD resulting from each topology were performed with a time scale of 12 ns, with exclusion of the first 2 ns from analysis. The optimization of the Gd^3+^ LJ parameters to reproduce the IOD was carried out with a production simulation of 50 ns, of which the first 2 ns were discarded from the analysis. The optimization of the Gd^3+^ LJ parameters for the reproduction of the τ_m_ was made with simulations which length was selected between 1 and 10 μs, to ensure a significant number of exchange events (at least 10 and typically close to 100), a compromise between the accuracy in the parameter under evaluation and the computational cost. Radial distribution function (RDF) profiles were obtained from these long simulations. Most analyses were done with GROMACS 2019 modules.^68^ The kinetics analysis of the water exchange process was done using scripts programmed with *Mathematica* v.12 (Wolfram). The H-bond analysis and the RDFs of specific water molecules with specific parts of the complex were done using scripts programmed with python v.3.9 using NumPy library v.1.22 (see section 2.3.2 for details).

For the normal-mode analysis, GROMACS 2019 was compiled with double precision as required for this calculation. Before this step, an energy minimization of the complex in a vacuum box was carried out with conjugated gradient algorithm with one steepest descendent step every 1000 steps, let to converge at machine precision.

### 2.2 Topologies

#### 2.2.1 General Considerations

The parametrization of the Ln complexes was based on the bonded model,^52,87^ as described in Appendix S.I.1 for the different force fields used (CGenFF compatible with CHARMM force fields,^88^ GAFF compatible with AMBER force fields,^89^ and GROMOS).^20^ Two strategies were followed, the so-called heuristic approach, and the use of the MCPB.py tool. The first approach was used to generate a molecular description with the CGenFF force field, whereas the second one led to an AMBER topology. Additionally, a united-atom topology using the GROMOS 54A7 force field was obtained. The missing parameters in the GROMOS 54A7 force field were converted from the topology that led to the best agreement with experimental observable τ_m_, bonds and angles, which in the present case was the topology that resulted from the MCPB procedure (TH, see further details below). Table 2 describes the various topologies studied in the present work and the nomenclature used throughout the text. The methodology employed in each situation was decided in order to be the most compatible possible with each force field chosen and with their recommendations. The overall simulation time was over 90 μs.

**Table 2:** Topologies Obtained and Compared in the Present Work and Corresponding Designations TA to TI as Used Throughout the Text

topology	basic approach	force field	ligand-only parameters	parameters involving Gd
TA	heuristic	CGenFF all-atom	CGenFF server	Gd^3+^ LJ parameters that reproduced IOD, from Li et al.^51^
TB	heuristic	CGenFF all-atom	CGenFF server	optimized from TA, to reproduce IOD
TC	heuristic	CGenFF all-atom	CGenFF server	Gd^3+^ LJ parameters that reproduced HFE, from Li et al.^51^
TD	heuristic	CGenFF all-atom	CGenFF server	optimized from TC, to reproduce τ_m_
TE	MCPB	GAFF all-atom	MCPB.py tool	Gd^3+^ LJ parameters that reproduced IOD, from Li et al.^51^
TF	MCPB	GAFF all-atom	MCPB.py tool	optimized from TE, to reproduce IOD
TG	MCPB	GAFF all-atom	MCPB.py tool	Gd^3+^ LJ parameters that reproduced HFE, from Li et al.^51^
TH	MCPB	GAFF all-atom	MCPB.py tool	optimized from TG, to reproduce τ_m_
TI	conversion	GROMOS54a7 united atom	ATB server	Converted from TH

#### 2.2.2 Heuristic Procedure

**Figure 2:**
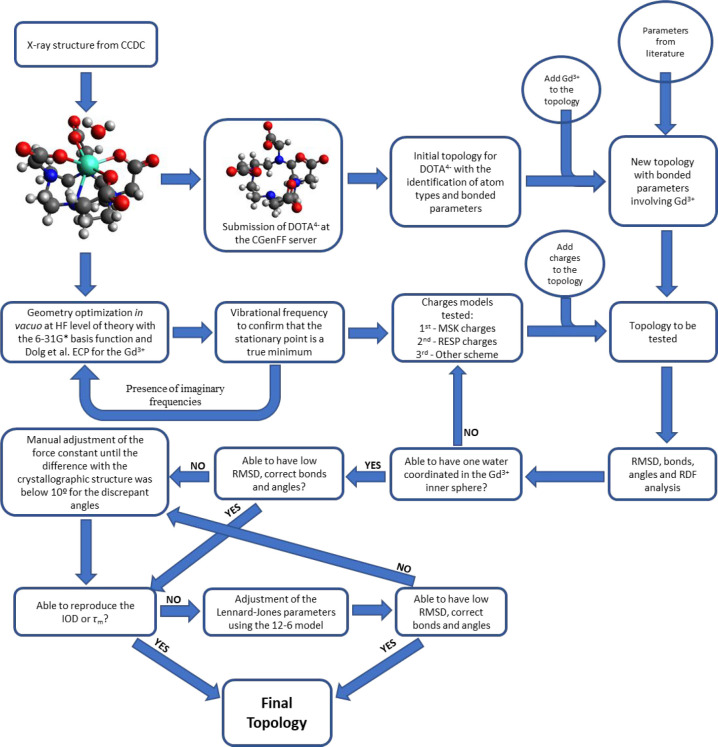
Parametrization scheme for the heuristic procedure.

The first attempt to parametrize [Gd(DOTA)]^−^ was based on a heuristic methodology, following the scheme illustrated in Figure 2, using the bonded model and adapting the parameters from the works of Henriques et al. and Dimelow et al.^35−37^ The initial parameters used are summarized in Table S1, with the corresponding naming of the atom types from those works in Figure S1.

The initial topology of [Gd(DOTA)]^−^ was obtained from the CGenFF server.^90,91^ As expected, the server does not recognize the Gd^3+^ ion; therefore, it was used only to obtain the atom types and bonded parameters for the ligand DOTA^4–^, using CGenFF version 4.1.^88^ The crystallographic structure of the complex was obtained from the Cambridge Crystallographic Data Center (CCDC)^92^ with the database identifier JOPJIH01 (or deposition number 1188960).^93^ The structure of the complex was edited with the Avogadro software,^71^ and subsequently, Open Babel software^73^ was used to obtain the complete numbering of the bonds, angles, and dihedrals. The 1,4-pairs were then added to the topology file. The force constants parameters for [Gd(DOTA)]^−^ involving Gd^3+^ ligand interaction were obtained from the literature,^35−37^ after separation of nonequivalent angles that were grouped in the original references (for which equilibrium values were taken from the X-ray crystallographic structure, taking into account the *C*_4_ symmetry of [Gd(DOTA)]^−^ in the SAP geometry). The dihedrals involving the Gd^3+^ ion were parametrized as 0 kJ/mol, and the initial van der Waals parameters were obtained from Li et al.’s work for hydrated Gd(III), with the TIP3P water model, that reproduced the IOD.^51^

Following the procedure of Henriques et al.,^35,36^ the determination of the atomic charges was carried out by *ab initio* calculations at HF theory level, keeping the capping water from the crystallographic structure. [Gd(DOTA)(H_2_O)]^−^ was fully optimized *in vacuo* without symmetry constraints, using the 6-31G* basis function set for the O, N, C, and H atoms. For Gd^3+^, the large-core relativistic effective core potential (LCRECP) of Dolg et al.^33,34^ and the associated (7s6p5d)/[5s4p3d]-GTO valence basis set were used, according to the methods employed by Henriques et al.^35,36^ The difference from those works to ours is the fact that they made single-point calculation directly from the crystallographic structure, whereas we performed a geometry optimization. In order to confirm that the resultant stationary point was a true minimum, vibrational frequency calculations were carried out to verify the absence of imaginary frequencies. With the final optimized structure, the electrostatic potential (ESP) was fitted, at the same level of theory and with the same basis function set, by means of the Merz–Kollman method.^94,95^ In this procedure we used the radius for the Gd^3+^ determined by Li et al., parametrized to reproduce the IOD with the TIP3P water model (1.623 Å).^51^ The charges obtained with the capping water were then averaged taking into consideration the *C*_4_ symmetry of [Gd(DOTA)]^−^. This averaging ensures that the charges are not conformation-dependent. At the end of this process, we performed a short simulation to test the topology. From RDFs of waters around the Gd^3+^ atom, it was concluded that no capping water remained coordinated to the Gd^3+^ (Figure S2A). In order to improve the quality of the topology, RESP charges were determined taking into consideration the *C*_4_ symmetry of [Gd(DOTA)]^−^ and freezing the mTIP3P charges to the capping water. RESP charges were determined using a two-step fitting procedure, as described by Cornell et al.^96^ In the first step, the charges of all atoms were allowed to vary according the *C*_4_ symmetry, avoiding charges dependent on the conformation, with a small restraint on the heavy atoms (0.0005) and no restraints on the hydrogen atoms. The second step refits the charges on the methylene groups (CH_2_) with a 0.001 restraint on the carbon atoms, and no restraints in the hydrogen of these groups, imposing again the *C*_4_ symmetry, avoiding charges dependent on the conformation, while the charges of the other heavy atoms (Gd, O, N, and the carbonyl C) were frozen at the values of the first fitting step. As observed by Henriques et al.,^35,36^ this strategy considerably improved the results, showing the presence of the inner-sphere water (Figure S2B).

Most structural parameters were well-reproduced with a low RMSD. Bond lengths established by the force field agreed with the crystallographic structure for bonds involving the Gd^3+^ ion. Deviations were found in the bonds involving hydrogen, since the force field establishes a longer bond than the X-ray structure. Most angles were well-reproduced compared to the crystallographic structure, except the Gd–O–C ones. This was solved by systematically increasing the force constant of the angle until the difference with the crystallographic structure was below 10°. The force constant thus obtained was 112.5 kcal/mol (Table S2), resulting in topology TA (see Table 2 for designation of the different topologies). This procedure was adopted every time there were discrepant angles. After the adjustment of the LJ parameters of the Gd^3+^ metal ion to reproduce IOD (see sections 2.3.1 and 3.1), the force constant of angle Gd–O–C was readjusted (Table S3), with the final topology (TB, Table 2) presented in Table S4, and the corresponding atom types named in Figure S3. In the procedure to fit LJ parameters of Gd^3+^ to reproduce τ_m_ (see sections 2.3.2 and 3.2), the initial guesses for LJ were made using the parameters that reproduce the HFE of the free Gd^3+^ ion from Li et al. parameters.^51^ This topology led to discrepant O–Gd–O and Gd–O–C angles, that were systematically readjusted as described above (Tables S5 and S6). This process resulted in topology TC. The changes in the topology after the fitting of the Gd^3+^ LJ parameters to reproduce τ_m_ resulted in topology TD and are presented in Table S7. In the latter topology, we observed that the Gd–O–C angle presented a difference above 10° but below 15° compared to the X-ray crystallographic structure. For the purpose of this work, the difference in this specific angle was considered acceptable and no further optimization was undertaken.

#### 2.2.3 MCPB Procedure

**Figure 3:**
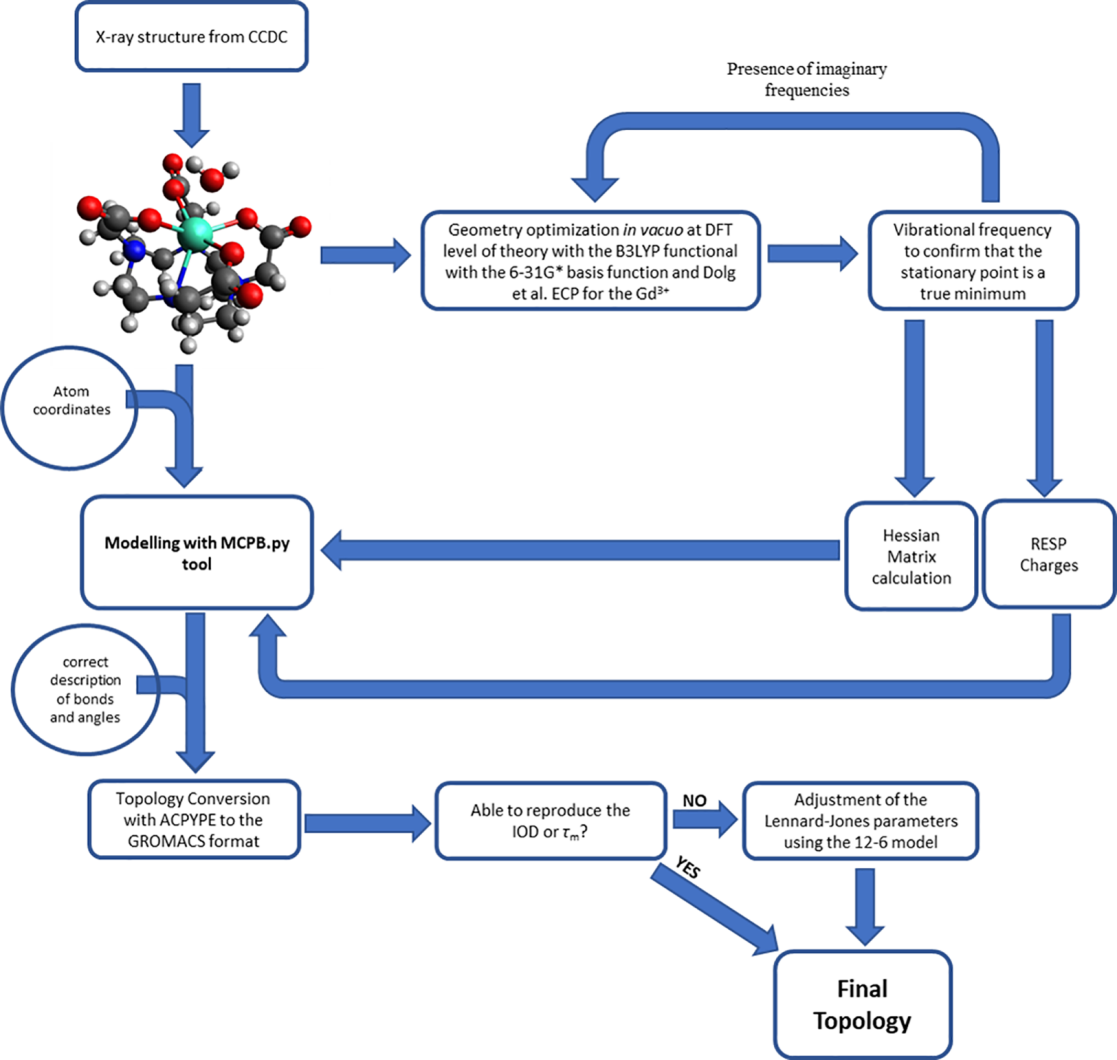
Parametrization scheme for the MCPB procedure.

The topologies obtained using the MCPB.py tool^52^ were derived following the scheme in Figure 3. The topology is based in the bonded model using the GAFF force field.^89^ The missing parameters from the GAFF force field involving the Gd^3+^ metal ion (force constants and bond and angle equilibrium values) were obtained based on a quantum mechanical calculation using the method proposed by Seminario et al.^56^ The procedure was adapted from Li et al.,^52^ and the topology obtained was converted to a GROMACS-compatible form using ACPYPE software.^75^ The [Gd(DOTA)]^−^ structure was also obtained from CCDC (see the Heuristic procedure, section 2.2.2). The optimization of the [Gd(DOTA)]^−^ structure was made in two steps *in vacuo*, with symmetry constraints to the *C*_4_ point group, using a quantum mechanical calculation at the DFT level of theory. The B3LYP functional, with the 6-31G* basis function set, was used for the O, N, C, and H atoms (as described in the examples presented by Li et al.^52^), while for Gd the LCRECP of Dolg et al. and the associated (7s6p5d)/[5s4p3d]-GTO valence basis set were employed.^33,34^ In the first step, the structure of the complex was modeled, to keep the N–Gd–O angle in the same acetate arm with an average angle equal to that from the crystallographic structure. The optimization of this structure was carried out with this angle frozen. This first step was necessary to ensure the SAP geometry with the closest possible twisted angle (ω). In the second step, angle restraints were removed, and the geometry was allowed to relax, keeping the symmetry constrained to the *C*_4_ point group. To confirm that the resultant stationary point was a true minimum, vibrational frequencies were calculated on the resultant geometry from the second optimization step to confirm the absence of imaginary frequencies. The ESP was calculated with the same level of theory and with the same basis function set. The density of points per unit area in ESP fitting was set using the overlay option of “iop(6/42) = 6”, employed by the authors who developed the GAFF force field, who found this option necessary to get reliable RESP partial charges, independent of molecular coordinates and orientation.^89^ The RESP charges were obtained with a two-step fit, in the same way as described in section 2.2.2. The initial LJ parameters for the Gd^3+^ metal ion were obtained from Li et al.'s work.^51^ This strategy led directly to a low RMSD value, MD averaged angles within 8° of those in the X-ray structure, and bonds in agreement with the input topology and X-ray structure (except those involving H, for reasons identical to those explained in section 2.2.2 above). Therefore, no adjustment of the force constant was required, resulting in topology TE (Table S8). The resultant topology after the optimization of Gd^3+^ LJ parameters to reproduce the IOD (see sections 2.3.1 and 3.1) is presented in Table S8 (TF) with the corresponding atom types naming in Figure S3. The topology with the LJ parameters that reproduce the HFE for the free Gd^3+^ ion from Li et al.^51^ is termed TG, and the one with LJ parameters fitted to reproduce the τ_m_ (see sections 2.3.2 and 3.2) is designated as TH.

#### 2.2.4 United-Atom Procedure

To create a united-atom topology, the GROMOS 54A7 force field was chosen. This force field is historically associated with the GROMACS software package and remains important in the simulation of biomolecules such as proteins and membrane lipids.^20^ The initial united-atom topology was obtained from the ATB web server.^97^ Similarly to the heuristic topology (see section 2.2.2), the Gd^3+^ ion was not recognized by ATB. For that reason, the initial topology was obtained for DOTA^4–^, in order to retrieve the atom types and bonded parameters for the ligand for use with GROMOS 54A7. Identification of the new bonded parameters indexes resulting from the addition of the Gd^3+^ metal ion was carried out as previously described (see section 2.2.2). The missing bonded and nonbonded parameters involving the Gd^3+^ were obtained by conversion from those resulting from the MCPB procedure, topology TH, which was the parametrization strategy that led to best agreement with experimental observables IOD, τ_m_, and structural properties (compared to the so-called heuristic approach, see below). Since the ATB server^97^ uses the Hessian QM analysis to derive missing parameters, this justifies the conversion of the missing parameters from MCPB procedure since they were also derived from Hessian-based calculations. For the bonded parameters involving the Gd^3+^ ion (bonds and angles), a conversion of the parameters obtained from the MCPB.py tools to values compatible with the GROMOS 54A7 force field was carried out (see Appendix S.I.2).^87^ The dihedrals involving the Gd^3+^ ion were also set at 0 kJ/mol. The atomic charges used in this topology were generated during the MCPB procedure but using the MK charges. The initial nonbonded parameters for Gd^3+^ were converted to values compatible with the GROMOS 54A7 force field, as described in Appendix S.I.2.

Most structural parameters were well-reproduced, with the exception of the Gd–O–C angle. This was solved, following the strategy from section 2.2.2, by systematically increasing the force constant until the difference with the crystallographic structure was below 10° (Table S9), resulting in topology TI. The final parameters of this topology are presented in Table S10, and the corresponding atom types naming and numbering are shown in Figure S4.

#### 2.2.5 Parameterization of the TSAP Isomer of [Gd(DOTA)]^−^ Using the MCPB Procedure

In addition to the molecular descriptions of the SAP isomer of [Gd(DOTA)]^−^obtained as described above, a topology for its TSAP counterpart was also developed. To this purpose, we used the optimized LJ parameters for Gd^3+^ of topology TH. Otherwise, the procedure follows closely that described in section 2.2.3. Specifically, the initial structure was taken from the crystallographic structure of [Ce(DOTA)]^−^ in the CCDC^92^ with the database identifier LUQBII (or deposition number 202157),^98^ which has a TSAP geometry. This complex was chosen since no crystallographic structure of the [Gd(DOTA)]^−^ in the TSAP geometry exists in the literature. The optimization of the [Gd(DOTA)]^−^ structure in the TSAP geometry was made in two steps, in the same way as in the MCPB procedure (see section 2.2.3), with *in vacuo* optimization, with symmetry constraint to the *C*_4_ point group and at the same level of theory and the same basis functions. In the first step, the N–Gd–O angle in the same acetate arm was also kept frozen during the optimization to keep the TSAP geometry with closest possible twisted angle. In the second step the geometry was allowed to relax, keeping the *C*_4_ symmetry. The remaining procedure is essentially the same with calculation of the RESP charges and the modeling with the MCPB.py tool. The LJ parameters for the Gd^3+^ were the ones obtained for the topology TH (see the “Results and Discussion” section for further detail). This strategy led directly to a low RMSD value, MD averaged angles within ∼10° of those obtained from the quantum mechanical calculation, and bonds in agreement with the input topology and quantum mechanical structure (except those involving H, for reasons identical to those explained in section 2.2.2 above). Therefore, no adjustment of the force constant was required, and the topology is presented in the Table S14.

### 2.3 Analysis

#### 2.3.1 IOD and CN Fitting

The optimization of the Gd^3+^ to water oxygen distance (IOD) and the number of water molecules in the first coordination sphere (coordination number of the Gd^3+^ in the inner sphere, CN) in order to reproduce the XRD experimental values for [Gd(DOTA)]^−^ (IOD = 0.249 nm, CN = 9^99^) was done by adjusting the nonbonded parameters of Gd^3+^. The Gd^3+^ Lennard-Jones parameters pair σ and ε were obtained following the approach used by Li et al. by scanning *R*_min_/2 values from 0.9 to 2.3 Å, with intervals of 0.1 Å, using the 12–6 LJ nonbonded model.^51^*R*_min_/2 was then converted to σ, which is the distance at which the LJ potential is equal to zero, using the following equation:
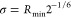
1

The ε values, the corresponding well depths of the LJ potential curve, were obtained for each *R*_min_/2 value using the noble gas curve proposed by Li et al.^49^ (ε in kcal/mol, *R*_min_/2 in Å units):
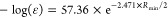
2The estimation of the Gd^3+^ LJ parameters was carried out by quadratic fitting of the IOD as a function of σ, followed by determination of the *R*_min_ (eq 1) and ε (eq 2) values that reproduced the experimental IOD.

#### 2.3.2 Mean Residence Time of the Water Molecules in the Inner Coordination Sphere (τ_m_) and in the Three Regions of the Outer Hydration Sphere, H-Bond Analysis, and RDFs of Specific Water Molecules with Specific Parts of the Complex

Calculations of the mean residence time (τ_m_) value (for refitting the Gd^3+^ LJ parameters to reproduce experimental values reported in the literature)^38,100,101^ for the heuristic (topology TD) and MCPB procedure (topology TH), and of the mean residence time for the water molecules in the three regions of the outer hydration sphere, only for the topology TH (see below sections 3.2 and 3.4), were carried out through the registration of events of water exchange, adapting the method developed by Filipe et al., with Mathematica v.12 (Wolfram) software.^67^ Briefly, the minimum and maximum distances between the Gd^3+^ ion and the oxygen of the water molecules in the different regions were calculated from the water oxygen RDF obtained for each simulation. The time at which each water molecule entered and left the distinct regions was identified, and the corresponding residence time (Δ*t*) was calculated. The rate constant was calculated from the best fit of a single exponential function to the distribution of Δ*t* values for the event considered. The position of the water molecules was also followed to track exchange events between the distinct regions and from those to bulk water. It was also possible to distinguish between a dissociative or associative mechanism of the coordinated water. In the refitting of the Gd^3+^ LJ parameters, the values determined by Li et al. that reproduce the hydration free energy^51^ were used as initial guesses, and then MD simulations were carried out, scanning different LJ parameters. The latter were obtained from different *R*_min_/2 and the corresponding ε from the noble gas curve,^49^ in the same way as in section 2.3.1. For these simulations, the water box was reduced, allowing for durations in the 1–10 μs range (see details in section 2.1). The estimation of the Gd^3+^ LJ parameters was then made through fitting of an exponential function to the τ_m_ versus σ variation. The optimized parameters were obtained by interpolation using the best-fitting exponential function. Finally, the system was simulated for 10 μs with this optimized parametrization for the validation of the final topology.

The RDFs of specific water molecules, according to their location in the outer hydration sphere (see Section 3.4), around specific parts of the [Gd(DOTA)]^−^ complex were obtained with scripts developed in python v 3.9 and with the module Numpy v 1.22. Initially, the distance from Gd^3+^ to all the water oxygens was calculated, as well as the distances between specific parts of the complex (namely, the coordinated and noncoordinated oxygens and the nitrogens) and all water oxygens, with the gmx distance module. The programmed script combined the data between the two distances and counted the frequency with a bin size of 0.01 nm. This analysis was carried out with topology TH for the first microsecond of the 10 μs trajectory, with exclusion of the initial 2 ns from the analysis.

For the analysis of the H-bonds, GROMACS has the gmx hbond module. However, this module only calculates the total H-bonds in each output frame and could not differentiate if a water molecule was in first hydration layer, intermediate region, or outer hydration layer or the possibility to identify the occurrence of two H-bonds involving a single water molecule, and for that reason, it was necessary to program our own scripts with python v.3.9 and with the module Numpy 1.22. The mathematical criteria for an H-bond were the defaults used by the GROMACS module. For example, for an H-bond involving an oxygen acceptor from [Gd(DOTA)]^−^ (P1), hydrogen from water (P2), and an oxygen donor from water (P3), the angle between the vectors  and  must be equal to or lower than 30°, and the distance between P1 and P3 must not exceed 0.35 nm. This analysis was done with the first microsecond of the 10 μs trajectory of the topology TH, excluding the initial 2 ns from the analysis, and dumping the coordinates from the trajectory with GROMACS module gmx traj. In order to validate our code, the total number of H-bonds in each output frame for that 1 μs was compared to the values given by GROMACS, and no discrepancies were found.

#### 2.3.3 Rotational Correlational Times (τ_R_)

Rotational autocorrelation functions were calculated for the tilt angles of the planes formed by the coordinated oxygens and nitrogens of [Gd(DOTA)]^−^, as well as of the vectors formed between the Gd^3+^ and the oxygen, and between the Gd^3+^ and the hydrogens of the capping water. From these functions, τ_R_ values were then obtained by exponential fitting. The rotational correlation times of the vectors between the Gd^3+^ and the capping water were obtained by averaging from 6 different waters randomly chosen that had coordinated with the Gd^3+^ from the TH simulation during the 10 μs. The τ_R_ was calculated using the second-rank Legendre polynomials (P2).^102^

## 3 Results and Discussion

### 3.1 Calculation and Optimization of IOD and CN

The analysis of the initial topology obtained from the so-called heuristic procedure using Li et al.’s Gd^3+^ nonbonded parameters^51^ (TA; see Table 3 for IOD, CN, and RMSD) shows that most structural parameters were well-reproduced with low RMSD and a correct description of bonds, angles, and improper dihedrals. A single water molecule is found in the first coordination sphere completing the 9-coordination sphere (CN = 9), in accordance with experimental data. However, some differences were observed for the IOD value obtained from the first maximum of the water oxygen RDF. For topology TA, IOD = 0.256 nm is recovered, which is higher than the experimental value of 0.249 nm.^99^

**Table 3:** Analysis Results of the RMSD, IOD, CN, and τ_m_ for Different Topologies^a^

topology	charges/Optimization	Gd^3+^ nonbonded parameters	Gd^3+^*R*_min_/2 (Å)	RMSD average/nm (standard deviation)	IOD (nm)	CN	τ_m_ (ns)
TA	RESP/HF-6-31G*	σ = 0.289 nm	1.623	0.0402 (0.004)	0.256	8.98	0.165
		ε = 0.382 kJ/mol					
TB		σ = 0.281 nm	1.579	0.0400 (0.004)	0.247	8.99	0.510
		ε = 0.289 kJ/mol					
TC		σ = 0.266 nm	1.495	0.0398 (0.004)	0.234	9.00	8.29
		ε = 0.157 kJ/mol					
TD		σ = 0.251 nm	1.410	0.0393 (0.004)	0.221	9.00	335
		ε = 0.0730kJ/mol					
TE	RESP/DFT-6-31G*	σ = 0.289 nm	1.623	0.0355 (0.007)	0.253	9.00	20.3
		ε = 0.382 kJ/mol					
TF		σ = 0.286 nm ε = 0.340 kJ/mol	1.604	0.0354 (0.007)	0.249	9.00	26.9
TG		σ = 0.266 nm	1.495	0.0353 (0.007)	0.229	9.00	b
		ε = 0.157 kJ/mol					
**TH**		**σ = 0.277 nm**	**1.554**	0.0350 (0.007)	**0.240**	**9.00**	**197**
		**ε = 0.244**kJ/mol					
TI	MK/DFT-6-31G*	C^6^ = 4.40 × 10^–4^ kJ mol^–1^ nm^6^	1.554	0.0461 (0.005)	0.257	1.00	3.88
		C^12^ = 1.98 × 10^–7^ kJ mol^–1^ nm^12^					
experimental values			1.107,^c^ 1.447,^d^ 1.746^e^		0.249^f^	1.00^f^	244^g^

a The final and recommended topology, TH, is in bold.

b No events observed in a simulation of 1 μs.

c Ionic radius.^103^

d UATM radius.^104^

e PCM radius.^105^

f See ref (99).

g See ref (38) and section 3.2 for discussion of experimental τ_m_ values.

**Figure 4:**
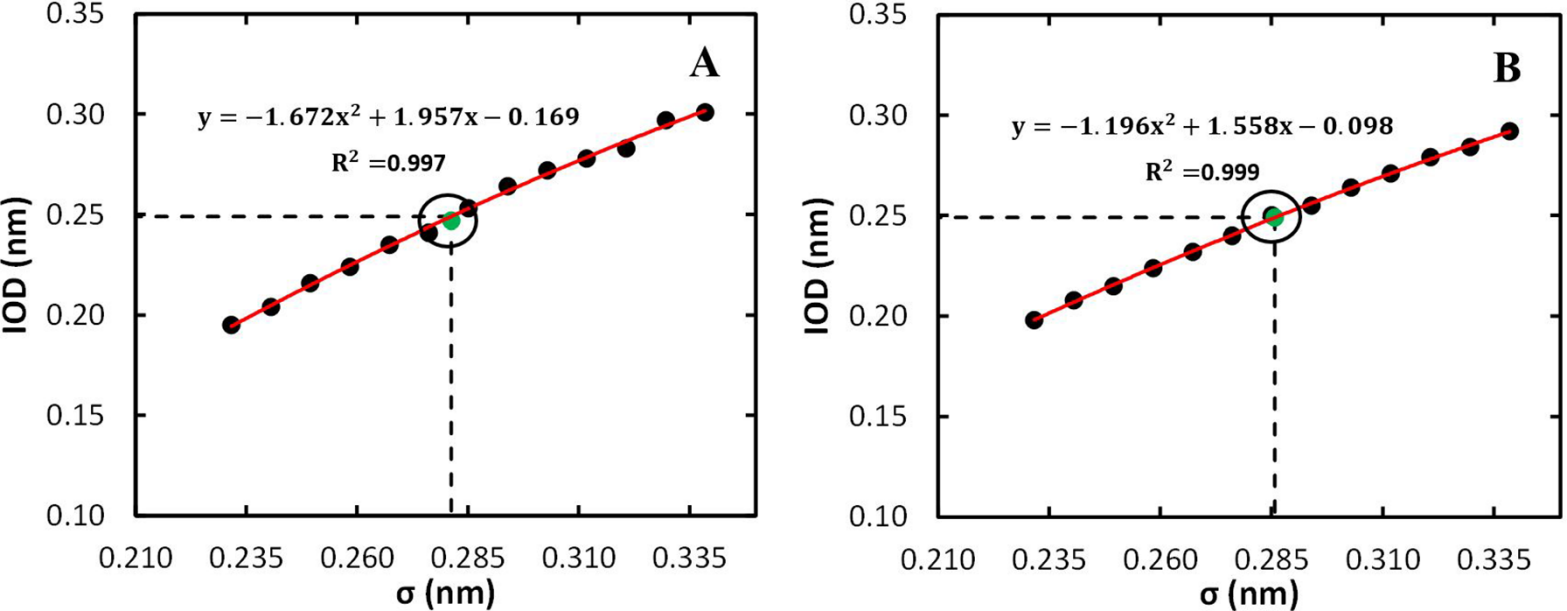
Fitting of the Gd^3+^ nonbonded parameters using the 12–6 LJ model to reproduce the experimental IOD for the heuristic topology (A) and for the MCPB procedure (B). The dashed horizontal line indicates the experimental IOD,^99^ and the vertical dashed line represents the optimized σ.

To address this issue, we employed the method described in section 2.3.1, by changing the value of Gd^3+^*R*_min_/2. Table S11 displays IOD and CN values obtained for each considered Gd^3+^*R*_min_/2, whereas Figure 4A depicts the best fit of a quadratic function to the IOD dependence with σ. Solving for the experimental IOD led to σ = 0.281 nm. After the correct adjustment of discrepant angles, this topology led to a small variation in IOD (0.247 nm), which remained close to the experimental value (topology TB, Tables S11 and 3).

For the MCPB procedure, the initially obtained topology (TE), with the Gd^3+^ nonbonded parameters obtained from Li et al.,^51^ was also able to reproduce well the different structural properties, except the IOD value that was slightly larger (0.253 nm), although it was closer than that obtained with the heuristic approach. Refitting of the Gd^3+^ nonbonded parameters, as described in section 2.3.1, was also carried out (Table S11 and Figure 4B). The new Gd^3+^ nonbonded parameters obtained were able to reproduce the experimental IOD (0.249 nm), while performing equally well regarding the other analyzed properties (TF).

The united-atom topology (TI) was obtained following the procedure detailed in section 2.2.4, leading to the correct description of the bond, angles, and improper dihedrals, except one angle that was further adjusted (see section 2.2.4), and the IOD (0.257 nm).

These results show that the final optimized topologies for the different approaches (TB and TF, for the heuristic and MCPB, respectively) were able to reproduce all experimental structural parameters, including the IOD.

**Figure 5:**
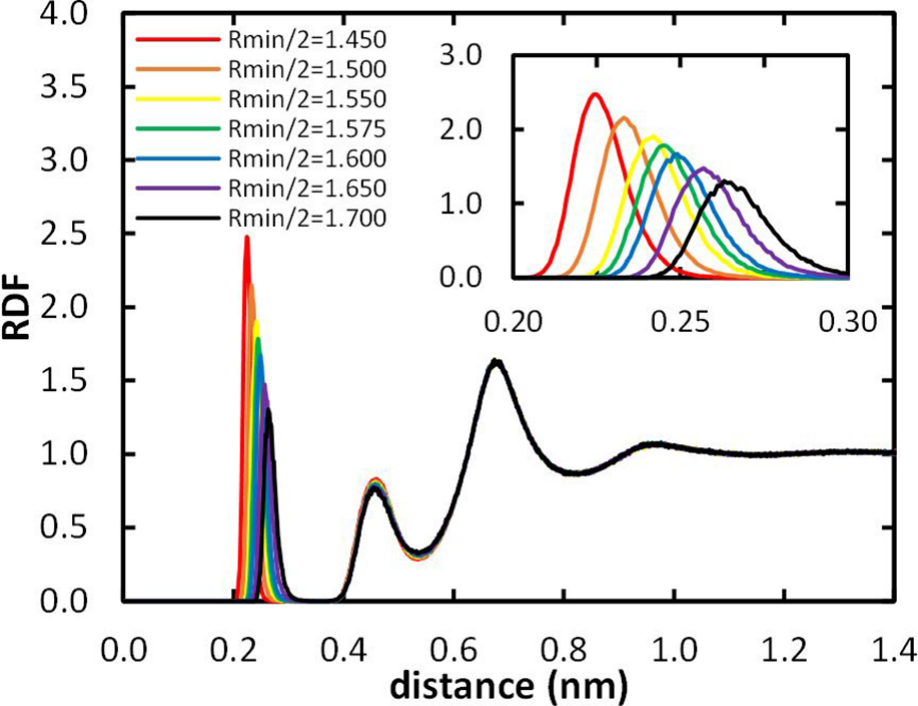
Gd^3+^–O_water_ radial distribution function for [Gd(DOTA)]^−^ for the MCPB procedure as a function of Gd^3+^*R*_min_/2 and zoomed in the 0.2–0.3 nm abscissa range.

For both the Heuristic and MCPB optimization procedures described in the “Methods” section, we verified that, by changing the Gd^3+^ LJ σ parameter (or, equivalently, *R*_min_/2), there is great sensitivity regarding the location of the water molecule in the inner coordination sphere. This becomes clear by observing the displacement of the first peak of the RDFs of water oxygen around Gd^3+^ (Figure 5). Shorter Gd^3+^*R*_min_/2 values lead to stronger Gd–O_water_ interaction, resulting in a lower IOD value, taken from the maximum of this peak. For longer distances, other peaks are observed in the RDFs, corresponding to other hydration layers. At variance with the inner coordination sphere, these peaks are insensitive to the Gd^3+^*R*_min_/2 and ε values used in the simulation, probably because water molecules in these locations do not interact directly with the metal ion, but with other atoms in the complex instead.

### 3.2 Calculation and Optimization of Water Exchange Kinetics Parameters

The parametrization strategies employed by several authors to model metal ions only consider the IOD of the complex or the HFE of the free metal ion and have difficulties with only one set of parameters reproducing both experimental targets. The solutions proposed by some authors are either based on polarizable force fields, with the consequent high computational cost reducing their applicability or employing alternative potential functions, such as in the work of Li et al. who proposed using the 12–6–4 LJ potential,^51^ which most MD packages do not support. In order to have a fully optimized topology, we believe that it is also necessary to consider dynamic properties, namely, the characteristic lifetime for the exchange of coordination water molecule (τ_m_), maintaining the IOD value as close as possible to the experimental value, with the same set of parameters. The value of τ_m_ was calculated from long simulations where a significant number of water exchange events were observed, following the procedure described in section 2.3.2. This value was compared with the original experimental determination of Powell et al. (244 ns).^38^ It should be noted that the experimental estimation of τ_m_ carries significant uncertainty, and both lower (185 ns)^101^ and higher (261 ns)^100^ values have been more recently reported. Moreover, these values concern the complex in solution, including contributions from the form simulated here (SAP), as well as the minor TSAP isomer (<20% prevalence), for which slower and faster exchange kinetics have been reported, respectively. In fact, the estimated values via deconvolutions of ^17^O NMR of the mixture of [Gd(DOTA)]^−^ isomers found a value of 360 ns for the SAP isomer,^100^ while in complexes constrained in the SAP conformation the τ_m_ found was 70 ns.^106^ For these reasons, we find that it is actually hard to select particular values of SAP τ_m_ within the 100–400 ns range as more exact than others, and therefore we consider that any values recovered in this range are acceptable for our purposes. As a reference value in the optimization of the Gd^3+^ LJ parameters, we considered the original experimental determination by Powell et al.,^38^ which is in the center of the expected interval of τ_m_ values.

**Figure 6:**
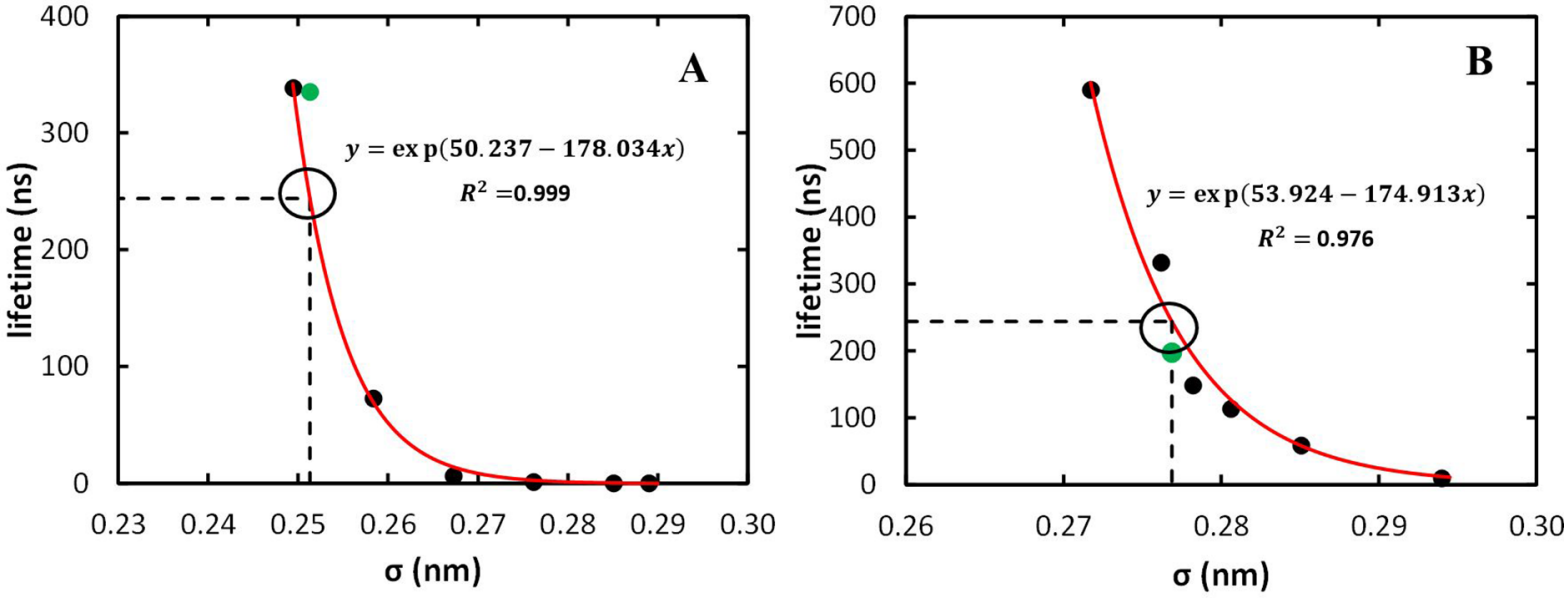
Adjustment of the Gd^3+^ nonbonded parameters using the 12–6 LJ model to reproduce the τ_m_ for the heuristic topology (A) and the MCPB procedure (B). The black dots correspond to τ_m_ values calculated from systematic variation of Gd^3+^*R*_min_/2. The best fit to these points is shown in red. Interpolation of the experimental τ_m_ with this curve is illustrated by the dashed lines. The green dot corresponds to the τ_m_ obtained in the validation run, carried out with the optimized parameters.

The optimization of the structural parameters to describe the IOD led to a better agreement with the experimental value of τ_m_, but it still underestimates τ_m_ for all considered approaches, namely, 0.51 and 26.9 ns for the heuristic and MCPB, respectively, compared to the experimental value of 244 ns (Table 3 and Figure S5).^38^ To address this issue, a second round of topology refinement was carried out. The LJ values obtained by Li et al.^51^ that reproduce the hydration free energy of hydrated Gd^3+^ were used as initial guesses (TC and TG for the heuristic and MCPB approaches, respectively). The smaller values of Gd^3+^*R*_min_/2 in these topologies led to smaller values of IODs (lower than the experimental value), and consequently a stronger Gd-capping water interaction, resulting in slower exchange kinetics. The values obtained are however not in agreement with the experimental value of τ_m_ = 244 ns, being smaller when following the heuristic approach (τ_m_ = 8.29 ns) and larger when the MCPB approach is followed (no events were observed in a 1 μs simulation). Therefore, for both heuristic and MCPB approaches, Gd^3+^*R*_min_/2 was systematically varied, leading to different LJ parameters as described in Section 2.3.2. For each (σ, ε) pair, τ_m_ was calculated and the results are shown in Table S12. The dependence of τ_m_ with σ was well described by an exponential function, and the best fit was used to obtain the optimized parameters (Figure 6). Finally, the resulting topologies (TD and TH for the heuristic and MCPB procedures, respectively) were simulated for 10 μs. The value obtained with the heuristic procedure (TD) deviates somewhat from the expected, which highlights the large uncertainty resulting from the low number of exchange events observed. Nevertheless, both topologies led to τ_m_ values of the same order of magnitude and within the range of acceptable experimental values (335 and 197 ns for the heuristic (TD) and MCPB (TH) topologies, respectively; see Table 3).

### 3.3 Topology Comparison

The main goal of this work is to find the best possible description of the lanthanide (III) complex that is simple enough for its use in simulation of complex systems, such as membranes and proteins. As explained in the introduction section, we focus on unpolarizable bonded models.

**Figure 7:**
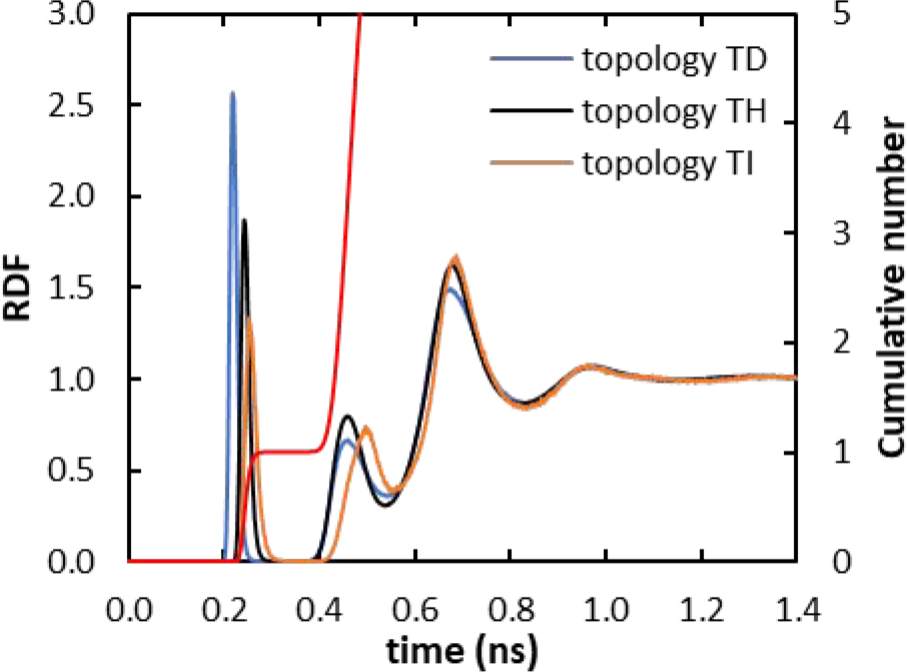
Comparison of Gd^3+^–O_water_ radial distribution functions for [Gd(DOTA)]^−^ for the topologies TD, TH, and TI, with the respective integration of the TH curve (red curve).

After the optimization of the topologies from the heuristic approach (TD)^35−37^ and MCPB.py procedure (TH), it is possible to see that the refined topologies reproduced well the X-ray crystallographic structure with low RMSD (Figure S6) and IOD close to that obtained from XRD,^99^ with the correct number of water molecules coordinated to Gd^3+^ (Table 3, Figure 7). Figure 7 shows that the different topologies also lead to similar distributions of water molecules around the Gd^3+^ ion. Although the first peak shows the variation in IOD associated with distinct LJ parameters for the Gd^3+^ in the different topologies, the remaining peaks of the RDF are similar for TD and TH.

As further verification of the quality of the topologies, the vibrational frequencies were calculated by normal-mode analysis and compared to those obtained by the DFT level of theory. No significant changes were verified, either among the different heuristic-based topologies or among the MCPB-based ones (data not shown). The comparison of the normal modes calculated based on DFT and MM methods (Figure S7) shows good agreement in both approaches. The linear fitting of the DFT and MM frequencies using the different topologies (TD and TH) show a *R*^2^ value for the fitting close to ∼1.00 for both topologies (Figure S8). This accordance was expected for the topology obtained from the MCPB procedure, since it uses the Seminario method^52,56^ which is based on the determination of force constants from the Hessian matrix, but vibrational frequencies were also reproduced using the heuristic procedure. The only discrepant vibrational frequencies in both topologies were the highest ones (>3000 cm^–1^). This may be due to the force constant in the bonds involving hydrogens, as shown in the works of Lin et al.^107^ and also noted by Li et al.^52^ Lin et al. were able to optimize these parameters, but they pointed out that most popular force fields use constraints in the hydrogen bonds like the SHAKE approximation,^107^ or, in our case, the LINCS algorithm. Thus, these high-frequency vibrations will not be an issue. This clearly shows the good quality of our topologies.

As expected, the topologies initially adjusted to reproduce the IOD (TB and TF) have negligible error in this parameter. However, when comparing the exchange lifetime of the water in the inner coordination sphere (τ_m_), it is observed that the use of parameters that reproduce the IOD leads to gross underestimation for the heuristic topology (almost 3 orders of magnitude). At variance, the topology obtained from the MCPB procedure underestimates τ_m_ by only 1 order of magnitude. Further adjustment of the Gd^3+^ nonbonded parameters to reproduce τ_m_, leads to values within 40% of the experimental value. This optimization required a decrease in Gd^3+^*R*_min_/2, which leads to an underestimation of IOD. However, this deviation is small, 11% for the heuristic procedure and 3% for the MCPB procedure. In this way, the adjusted Gd^3+^ nonbonded parameters are able to reproduce simultaneously a dynamical property (τ_m_) and IOD with little error, especially in the case of the MCPB procedure with topology TH.

The estimated Gd^3+^*R*_min_/2 values are 1.41 and 1.55 Å for topologies TD and TH, respectively. The “crystal” ionic radius, determined by Shannon for Gd^3+^ with a coordination number of 9, was 1.107 Å^103^ or 1.105 Å determined by D’Angelo et al.,^47^ 1.00 Å by Marcus,^108^ or 1.01 Å by Heyrovska.^109^ The van der Waals radius is expected to be larger. However, there is no agreement on how much larger the van der Waals radius of Gd^3+^ should be. For example, in the works of Cosentino et al.^104^ and Regueiro-Figueroa et al.,^105^ the solute cavities are constructed by parametrizing the Ln^3+^ radius using the polarizable continuum model (PCM), obtaining values for Gd^3+^ of 1.447 and 1.746 Å, respectively. There is also the example of the work of Migliorati et al. that had determined the Gd^3+^ LJ parameters with an *R*_min_/2 of 1.72 Å^46^ or the work of Yerly et al., who used a *R*_min_/2 of 1.80 Å.^44^ The radius recovered for the heuristic method (TD) is below the lower limit of this range of values, while in the case of the MCPB method (TH), Gd^3+^*R*_min_/2 is inside the range of those values. Following the procedure suggested by D’Angelo et al.,^47^ subtracting the radius of water (1.35 Å) from the IOD distance of TH, one obtains a ionic radius of 1.05 Å, close to the above-mentioned ionic radii.

This study shows a superior performance of the MCPB method compared to the heuristic procedure. One of the reasons for this is the fact that the MCPB uses quantum mechanical calculations to obtain a Hessian matrix, which is used to determine the force constant for bonds and angles, ensuring an intrinsically corrected structure. Another advantage of the MCPB procedure is that it is less laborious than the heuristic procedure (see Figures 2 and 3 for comparison), with fewer steps for the construction of the topology and with fewer optimization steps.

Further evidence from this study is that even though Li et al. optimized separately a set of nonbonded parameters that reproduced well the IOD or the HFE properties^51^ for the free ion, when the ion is complexed with a ligand and using the bonded model, an adjustment of their nonbonded parameters is required. As noted by Li et al.^51^ with the 12–6 LJ model, it is difficult to reproduce multiple properties simultaneously. However, with the adjustment of the parameters, we were able to reproduce IOD and τ_m_ simultaneously, with lower error in the IOD with the MCPB procedure (TH) compared to the heuristic approach (TD).

For the reasons pointed out previously, we chose the parameters obtained from the MCPB procedure (TH) to obtain a united-atom topology by conversion. After some optimization in one angle, the structural properties were well-reproduced with low RMSD (Figure S6 and Table 3), with low error on IOD (Figure 7 and Table 3) and a τ_m_ shorter by 2 orders of magnitude (Figure S5 and Table 3). Since the optimized LJ parameters of Gd^3+^ were used, the difference is probably due to the ligand and water force fields. For this reason, we did not pursue further adjustments on the LJ of Gd^3+^ in attempt to reproduce the IOD or τ_m_. With this approach, it was shown that conversion of the parameters from the MCPB procedure to a united-atom topology using a GROMOS force field is possible. This could be useful in the context of reducing even more the computational cost of a simulation in comparison with the other two all-atom strategies detailed in the present work, allowing the study of complex systems with a larger time scales, at the cost of some inaccuracy.

### 3.4 Detailed Analysis of the Structure and Dynamics of the Optimized Topology

After selecting the topology TH as the most adequate one, a detailed analysis of the dynamics of the interactions between water and the [Gd(DOTA)]^−^ complex is described in this section. This includes information regarding the mechanism of water exchange as well as the number and dynamics of the water molecules in the outer hydration sphere.

**Figure 8:**
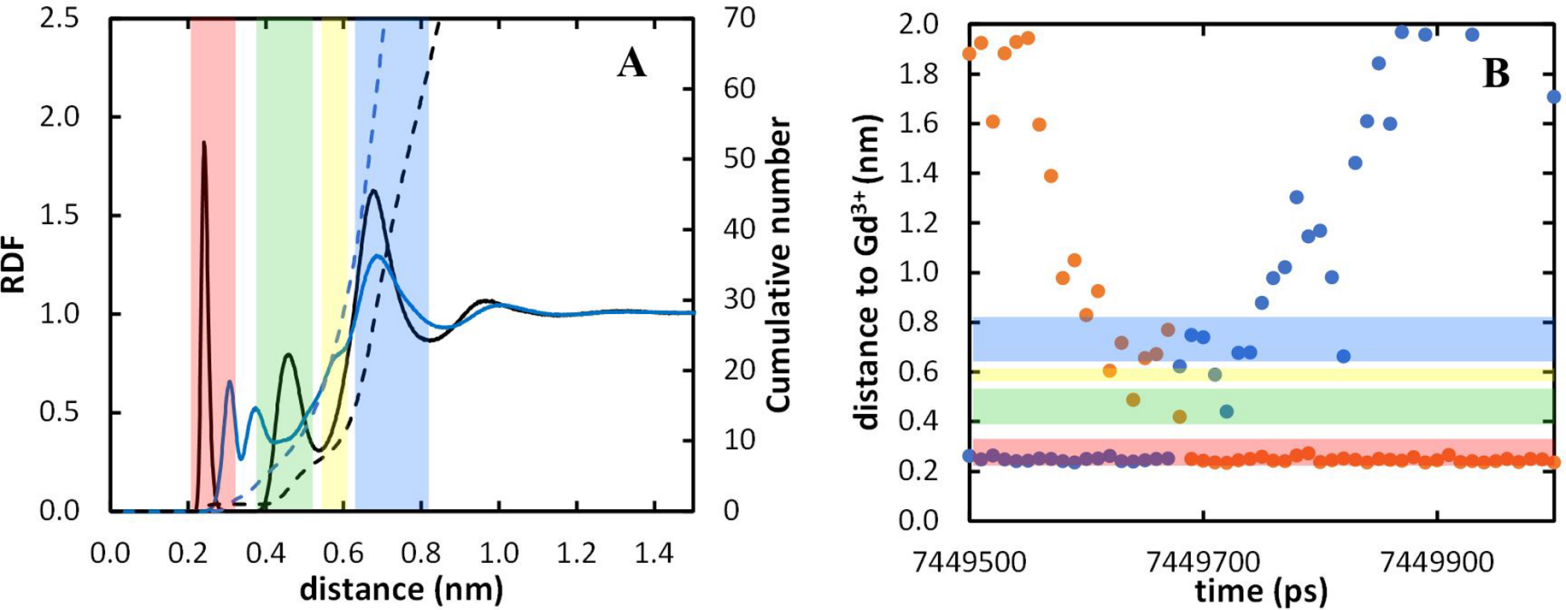
Gd^3+^–O_water_ (black line) and Gd^3+^–H_water_ (blue line) radial distribution functions for the [Gd(DOTA)]^−^ for the topology obtained from MCPB (TH) in a 10 μs simulation with the illustration of the cut-offs for the inner sphere (red bar), for the first hydration layer (green bar), the intermediate region (yellow bar), and the outer hydration layer (blue bar) used to study the kinetics (A), as illustrated in the panel B, where it is possible to observe a dissociative event in the inner sphere (blue and orange dots correspond to distinct water molecules, dissociating from/coordinating to the Gd^3+^ atom, respectively).

Figure 8A shows the Gd^3+^–O_water_ radial distribution function. Analysis of these results allows us to categorize the water surrounding the complex into two main regions: the inner coordination sphere, which contains only the water molecule closest to the lanthanide and corresponds to the first peak of the RDF marked with the red shaded region, and the outer hydration sphere, for a Gd^3+^–O_water_ distance between 0.3 and 0.8 nm. The outer hydration sphere is further subdivided into the first hydration layer (green shading), an intermediate region (yellow shading), and an outer hydration layer (blue shading). The integration of the RDF and the Gaussian distribution in Figure S9 show that there are on average 6 water molecules in the first hydration layer, 52 in the outer hydration layer, and 5.7 in the intermediate region (Table 4). It is relevant to note that although the average number of molecules is similar in the inner and intermediate hydration layers, the width of the distribution is much smaller in the former (Figure S9). This points toward more specific interactions established between the water molecules and the complex in the case of the inner hydration layer. Once the boundaries of the hydration regions were defined, the dynamics of each water molecule was followed.

**Table 4:** Analysis of the Different [Gd(DOTA)]^−^ Hydration Layers for Topology TH^a^

	*q*	water residence time in the different regions (τ)
inner sphere	1.0	197 ns
first hydration layer	6.0	18 ps
intermediate layer	5.7	10 ps
outer hydration layer	52.3	19 ps

a With Gd^3+^ van der Waals radius of 1.554 nm and the indication of the number of water molecules (*q*) and corresponding residence lifetime (τ).

During the 10 μs simulation time, 49 exchange events of water molecules in the inner coordination sphere were identified. Those correspond to distinct water molecules. In the inner coordination sphere two water molecules were never encountered simultaneously, in agreement with a dissociative mechanism, where the water from the inner sphere leaves this sphere and only after that a new molecule enters, as anticipated from the literature.^38^ When leaving the inner coordination sphere, half the water molecules stayed for at least 20 ps in the outer hydration sphere, while the other half moved directly to the bulk. A similar situation was observed for the new molecules entering the inner sphere, with 42% of the molecules coming directly from the bulk while the remaining stayed previously at least 20 ps in the outer hydration sphere. A representative exchange event is shown in Figure 8B. The water molecule initially in the inner coordination sphere moves into the outer hydration sphere where it stays for about 70 ps and from there moves into the bulk. In this exchange event, the water molecule that enters the inner coordination sphere came from the outer hydration sphere, where it had stayed for about 80 ps. Considering the exchange events involving the first hydration layer, almost 10^6^ were observed during the 10 μs simulation time. In these events, a significant fraction of the water molecules moved into the outer hydration layer (32%), whereas the remaining 68% move into the bulk in less than 20 ps. The outer hydration layer is a very dynamic region with nearly 10^7^ exchange events, 97% of which move to the bulk. All individual 1706 water molecules visited the three different regions of the outer hydration sphere. Figure S10 shows a Gaussian-shaped distribution of the frequency of exchange events per number of water molecules in the different regions of the outer sphere.

**Figure 9:**
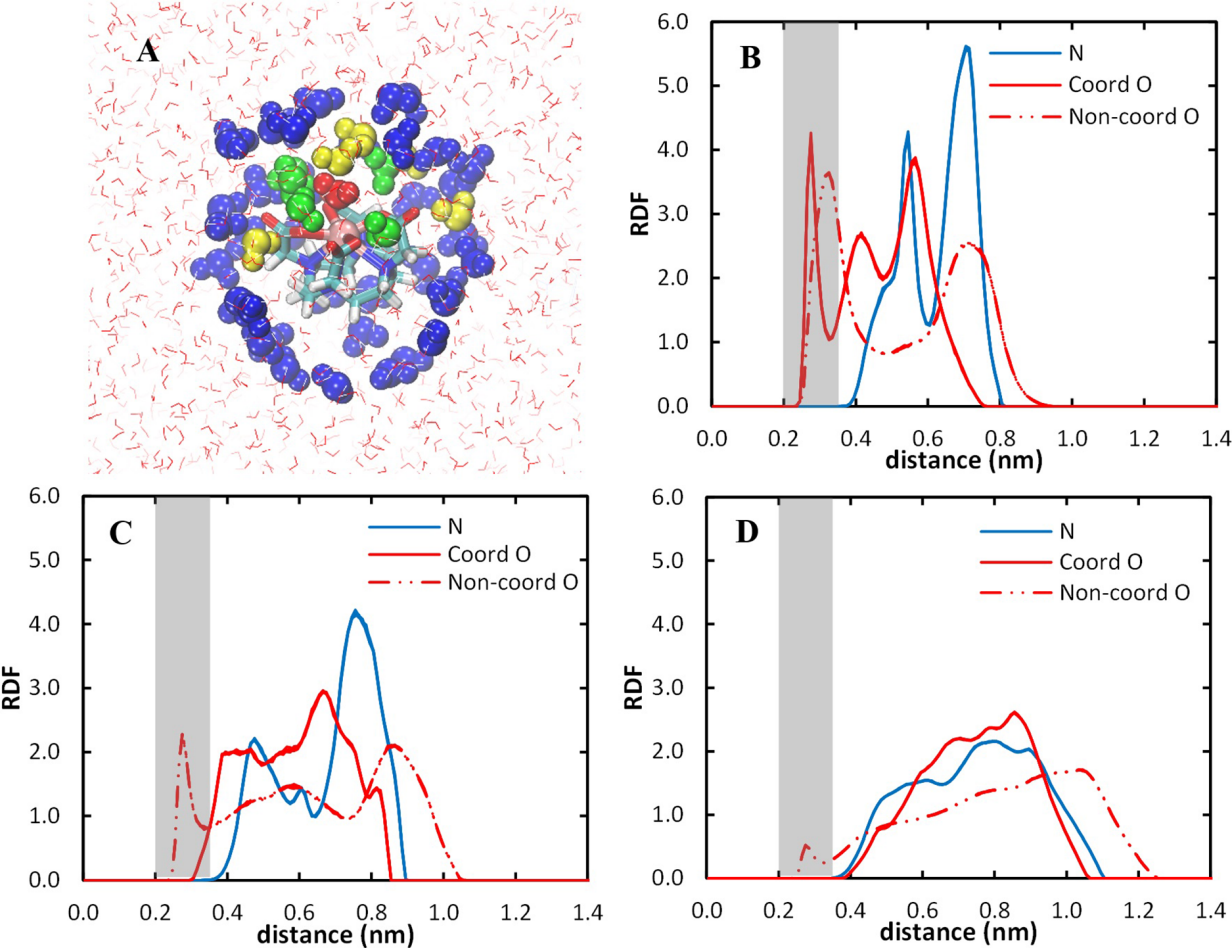
Snapshot of the system at 941 800 ps showing the organizations of the waters in different layers: inner-sphere water (red), first hydration layer (green), intermediate region (yellow), and outer hydration layer (blue). For better visualization, some water molecules were removed from this latter region (A). RDF of the water oxygens in the first hydration layer (B), intermediate region (C), and outer layer (D) around different parts of [Gd(DOTA)]^−^, namely, nitrogens (blue), Gd^3+^-coordinated (red), and non-Gd^3+^-coordinated oxygens (dotted and dashed red line). In panels B–D, each curve corresponds to the overlap of the RDF of the four individual curves from the chemically equivalent atoms in the complex. These figures show a gray band corresponding to the cut-offs for possible H-bond formation between the water molecule and each specific part of the complex.

The snapshots in Figures 9A and S11 show the location of the water molecules in the different regions. The water in the inner coordination sphere is shown in red, surrounded by the water molecules in the first hydration layer (shown in green). The outer hydration layer shown in blue surrounds completely the complex, unlike the first hydration layer (green molecules) that is located only in the hydrophilic part of the complex. The water molecules shown in yellow represent the intermediate region between the first and outer hydration layers and are also located in the hydrophilic part of the complex. They differ from the molecules in the first hydration layer by having slightly more external locations.

In Figure 9B–D, the RDFs illustrate in a graphical and quantitative way the considerations taken from the snapshots (Figures 9A and S11). In each curve, the first peak of the RDFs gives information about how close the water molecules in each layer of the outer sphere are to specific parts of the complex, namely, the Gd^3+^-coordinated and non-Gd^3+^-coordinated oxygens and nitrogens of DOTA. The gray bar gives the cut-offs for possible formation of H-bonds between the water H-bond donor atoms and H-bond acceptor atoms of the complex. Complete overlap of the RDFs between chemical equivalent atoms is observed in Figure 9, as expected from the *C*_4_ symmetry of the complex. Figure 9B,C shows that the water molecules from the first and intermediate hydration layers are closer to the coordinated and noncoordinated oxygens than to the nitrogens of [Gd(DOTA)]^−^, indicating a localization in the hydrophilic part of complex. The closest peak appears with the coordinated oxygens for water molecules in the first hydration layer and with the noncoordinated oxygens for water molecules in the intermediate hydration layer. The RDF plots indicate possible H-bond formation between water molecules in the first hydration layer and both the coordinated and noncoordinated oxygen atoms, while those in the intermediate region establish H-bonds with the noncoordinated oxygens only. Relative to the water molecules in the outer hydration layer, in agreement with the snapshots that show that this layer surrounds all the complex, the RDFs show similarities in the distribution of the water molecules around the more internal coordinated oxygens and nitrogens of [Gd(DOTA)]^−^. This figure shows a short-distance peak, indicating proximity of some water molecules of the outer hydration layer to the more external noncoordinated oxygens. In fact, this peak appears in the zone of possible H-bonding (Figure 9D).

**Figure 10:**
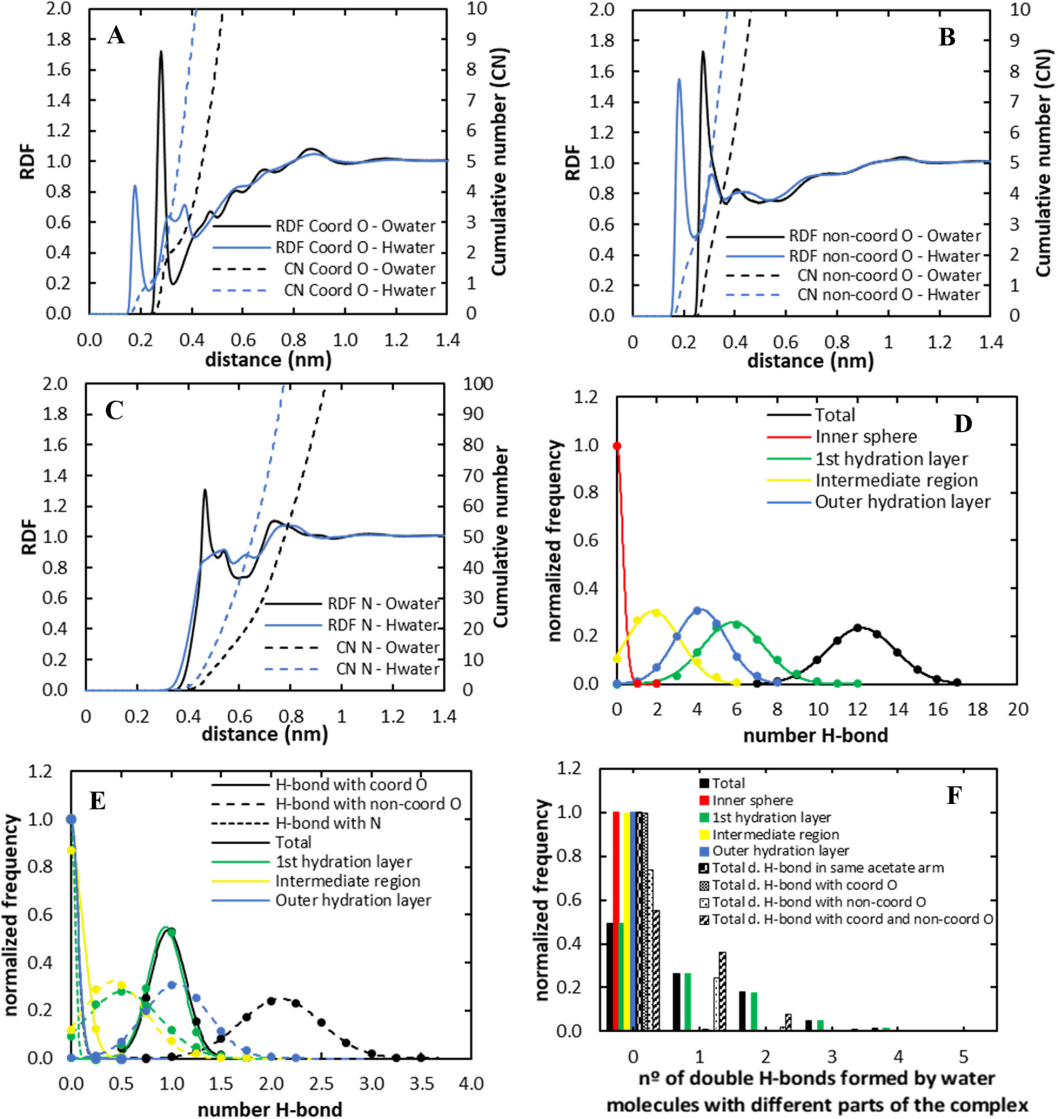
O_acetate_–O_water_ (black line) and O_acetate_–H_water_ (blue line) RDFs for waters around the oxygens of the acetate arm coordinated (A) and not coordinated to Gd^3+^ (B) for topology TH. Each plot includes the integration of the RDFs to obtain the cumulative numbers (dashed lines with respective color). N–O_water_ (black line) and N–H_water_ (blue line) RDF and the corresponding integration (dashed lines with the corresponding color) (C). Distribution of the total number of H-bonds formed between all the water molecules and the complex, in each output frame (black line), distribution of H-bonds between the complex, and the water molecules in the inner sphere (red line), first hydration layer (green line), intermediate region (yellow line) and the outer hydration layer (blue line) (D). Number of H-bonds formed per atom and per output frame between the four-coordinated (solid line) and noncoordinated oxygens (dashed line) and the four nitrogens (dotted line) of [Gd(DOTA)]^−^ with the total of water molecules (black), only the water molecules in the first hydration layer (green), intermediate region (yellow), and outer hydration layer (blue) (E). In plots D and E, the points representing the normalized frequencies were described by Gaussian functions (lines). Number of double H-bonds formed between one water molecule to two different regions of the complex, with identification of the locations of the involved water molecules (F).

A closer look at the Gd–O_water_ and Gd–H_water_ RDFs (Figure 8A) shows that the oxygen of the inner coordination sphere water is closer to (and directly interacting with) Gd^3+^ than the hydrogen atoms of the same water molecule, with the integration of the first peak of Gd–H_water_ RDF equal to two hydrogen atoms, as expected. The opposite is observed in the first hydration layer, where the water hydrogens are closer to Gd^3+^ than the oxygens, pointing to possible interaction of the water H atoms with H-bonding acceptor atoms of the complex. The water molecules in the intermediate region have a more diffuse orientation. This is observed to a larger extent with the water molecules in the outer hydration layer, which are more distant from the Gd^3+^ ion and display an essentially random orientation toward the latter. A further investigation, with the acquisition of the O_acetate_–O_water_ and O_acetate_–H_water_ RDFs (Figure 10A,B), shows that the water molecules interact with the oxygens of the acetate arms, leading to strong peaks indicative of H-bond formation. Comparing the hydrophilic hemisphere of [Gd(DOTA)]^−^ with its hydrophobic counterpart, the latter region shows lack of specific interactions involving water molecules, evidenced by the absence of peaks below 0.4 nm in the N–O_water_ and N–H_water_ RDFs (Figure 10C).

Analysis of the H-bond formation was done using the criteria described in section 2.3.2. On average, in each instant, there are 12.2(±1.0) H-bonds between water donors and [Gd(DOTA)]^−^ acceptors, with 0.0 (±0.3) coming from water molecules in the inner sphere, 5.8 (±1.5) coming from the first hydration layer, 1.8 (±1.3) from the intermediate region and 4.2 (±1.3) from the outer hydration layer (Figure 10D). Figure 10E shows that on average each noncoordinated oxygen forms 2.1 (±0.4) H-bonds with water molecules irrespective of their location, the coordinated oxygens form 1.0 (±0.2) H-bonds, while the nitrogens form 0.0 (±0.1) H-bonds. The difference in the number of H-bonds formed between the oxygens is expected due to steric effects, since the noncoordinated oxygen of each arm is more exposed to the solvent. Similar values were obtained by Borel et al.^42^ The lack of H-bonding to the complex nitrogens is unsurprising, because these atoms are clearly less electronegative than the oxygens, as well as more sterically hindered. Further investigation on the location of the water molecules forming H-bonds with different parts of the complex shows that almost all H-bonds to the coordinated oxygens come from water molecules in the first hydration layer. In contrast, H-bonds to the noncoordinated oxygens come from water molecules in different locations, with the highest frequency coming from those in the outer hydration layer (Figure 10E). In the density maps shown in Figure S12A, we can see that a high density of O_water_ is observed in the center, corresponding to the interaction of the inner-coordination-sphere water with the Gd^3+^ ion. In Figure S12B, the hydrogen density map shows a high density ring around the center (in green), resulting from axial rotation of the coordinated water molecule. At slightly longer distances from Gd^3+^, four locations with high density of H_water_ (blue regions) are also observed, corresponding to water molecules H-bonded to the acetate arms. Some enrichment of O_water_ is also apparent in the corresponding regions of Figure S12A. These figures illustrate the *C*_4_ symmetry of [Gd(DOTA)]^−^. The possibility of one water molecule forming two distinct H-bonds, with each hydrogen binding to acceptors in different regions of the complex, was also investigated (Figure 10F). It was found that on average 1.7 H-bonds (out of the 12.2 total) were formed from one single water, and only involving water molecules in the first hydration layer. Acceptors of these double bonds were either two noncoordinated oxygens or one coordinated and another noncoordinated oxygen. A negligible fraction of double bonds involved one water molecule and two coordinated oxygens, as well as two oxygens of the same acetate arm. This result evidences the presence of double H-bonding events, as hypothesized by Racow et al., who studied the complex [Gd(DOTA)]^−^ in gas phase by mass spectrometry. These authors found a greater entropically disfavored interaction between the water molecule in the first hydration layer and the complex, compared to the inner-sphere water molecule and the complex. It was hypothesized that this could be due to the formation of two H-bond between both hydrogens of one water molecule and oxygens in different acetate arms of the complex, that restrained the rotation of the water with the consequent entropic penalty.^110^ In a less detailed work by Borel et al., these authors also found, from their simulations, the occurrence of water molecules forming two H-bonds, with higher frequency than found in this work, but without mention of the localization of the water molecules. The differences observed may be due to the distinct parametrization, namely, the introduction of a covalent bond between the capping water and Gd^3+^.^42^ With this work, we show the occurrence of such double H-bond events in solution, although with a relatively low frequency. From all this analysis, one can conclude that the two distinct hemispheres in [Gd(DOTA)]^−^ (hydrophilic and hydrophobic regions) induce a very different organization of the surrounding waters, in agreement with the works of Borel et al.^42^ and Henriques et al.^36^

The kinetic analysis shows that the residence lifetime of the waters in the first hydration layer (τ_first hydration-layer_) is equal to 18.4 ± 0.1 ps and essentially the same for all topologies evaluated (Table S13). This shows that the dynamics of water molecules in this layer is not dependent on the *R*_min_/2 of Gd^3+^, as expected since the chelator DOTA^–^ is the responsible for the interaction of the water molecules in the various layers of the outer sphere. This is further proved by the overlap of the first hydration layer, intermediate region, and the outer hydration layer in the Gd^3+^–O_water_ RDFs across the tested Gd^3+^*R*_min_/2 values (Figure 5). This clearly indicates that, as discussed previously, other interactions such as formation of hydrogen bonds between the oxygen atoms of the acetate arms and the water molecules determine the average structure and dynamics of those layers. The value of τ_first-hydration-layer_ lies between those obtained by Henriques et al. (13.7 ps), who considered 20 water molecules in this region,^36^ and by Borel et al. (27.4 ps), who only considered a hydration number for this region of 4.3.^42^ In this work, intermediate values are observed for both distance and residence time. The kinetic analysis for the water molecules in the other regions of the outer hydration sphere was also analyzed for the final topology (TH). Water molecules in the intermediate region show a residence time of 10 ps, while for those in the outer hydration layer, a residence time of 19 ps was obtained (similar to that of the water molecules in the first hydration layer). The latter values show that the water molecules in the outer hydration layer do not interact extensively with the chelate, which can be understood by noting that only 4 out of 52 waters form H-bonds with the chelate, showing that these waters are mainly in self-diffusion. Regarding the other two regions, the interactions that occur between those water molecules and the complex prolong their residence time beyond expected from purely diffusional translation. This is in agreement with the results of the H-bond analysis, where the waters in the first hydration layer and intermediate region form 6 and 2 H-bonds out of the 6 and 5.7 waters in these regions, respectively. In conclusion, it is the overall geometry and charge distribution of the complex that mostly determines the first hydration layer, intermediate region, and outer hydration layer.

### 3.5 Calculation of the Water Rotational Correlation Times

One important parameter that governs water proton relaxivity in the presence of a Gd^3+^ chelate is the correlation time, which is influenced by the electronic relaxation time, rotational correlation times, and water exchange lifetime. The fastest process is the one that most influences the relaxivity. So far, we have analyzed the water exchange dynamics. The electronic relaxation time for the Gd^3+^ is slow compared to the other two processes at clinically important magnetic field strengths (≥1.5 T), and for that reason its influence on the relaxivity is not taken into consideration. The last important property is the rotational correlation time (), that in fact, for small contrast agents, is usually the fastest process. In this scenario, a longer  increases the proton relaxivity process, thus increasing the MRI sensitivity (for further detail see ref (5)). Yerly et al. remarked that different values of  should be considered, namely, those obtained from the vectors formed between Gd–O_water_ and Gd–H_water_, in opposition to the averaged  obtained from experimental techniques.^44,45^

Table 5 shows the different  values obtained from MD simulation with TH. A slightly shorter  was obtained from the Gd–H_water_ vector than from the Gd–O_water_ vector. The ratio (0.83) is within the range 0.65 ± 0.2, inferred from experimental work by Dunand et al.^111^ and is very similar to that obtained from simulation by Yerly et al. of 0.82.^45^ We also calculated rotational correlation times from the normal to the planes defined by the O_acetate_ atoms coordinated with Gd^3+^ and by the nitrogen atoms. The resulting values are virtually identical to that of the Gd–O_water_ vector. This shows that the oxygen of the capping water is an integral part of the coordinating sphere of Gd^3+^. In the work of Yerly et al., although they used the same water model (TIP3P), they suprisingly obtained values twice the value in this work, namely,  of 52, 51, and 41 ps for the polyhedron, vector Gd–O_water_, and vector Gd–H_water_, respectively.

**Table 5:** Calculated Second-Order Rotational Correlation Times for [Gd(DOTA)]^−^a

	τ_R_ (ps)
(Gd–O_water_) vector	23.8 (0.3)
(Gd–H_water_) vector	19.8 (0.3)
(O_acetate-coordinated_) planes	23.7
(N) planes	23.8
τ_R_Gd–H_water_/τ_R_Gd–O_water_	0.83
Gd–O_water_ (SPC/E water model)	54.5
experimental^38^	77

a Standard deviations are shown inside parentheses.

Table 5 shows a discrepancy by a factor of 3 between the experimental (77 ps)^38^ and the calculated rotational correlation times. One possible reason for this disagreement is the indirect procedure to obtain the experimental values from relaxation times. Additionally, it may also result from the well-documented underestimation of the viscosity of the TIP3P water model by a factor of 2.8, and the corresponding faster self-diffusion overestimation by a factor of 2.4.^112^ For that reason, we tested our topology with the SPC/E water model (which is not the standard model for the GAFF force field), which has higher viscosity.^112^ Unsurprisingly, the rotational correlation time obtained with the SPC/E model increased, approaching the experimental value.

In this work, our main focus was to develop simple methodologies to obtain a reliable topology model for contrast agents for use in more complex systems, such as those containing membranes, proteins, or nucleic acids. However, with our model, physicochemical properties like τ_m_ and τ_R_ can also be estimated in order to evaluate the efficiency of Gd^3+^-based complexes as contrast agents.

### 3.6 Applicability of the Optimized Gd^3+^ LJ Parameters to Other Gd^3+^ Chelates

In principle, the Gd^3+^ LJ parameters optimized here should apply to any Gd^3+^ ion, regardless of its coordination within a given complex. Individual details related to coordination should be directly reflected in the bonding parameters (bond lengths and angles) and in the Gd partial charge, not the LJ nonbonding ones. As pointed out in the Introduction, different LJ parameters often result from fitting to distinct experimental observables. Ultimately, we chose comparison with the experimental capping water residence time τ_m_ for validation of our definite topology TH, mostly because of its high sensitivity: relatively small changes in Gd^3+^ σ produce large changes in the calculated τ_m_. We obtained σ = 0.277 nm for topology TH, having started from the value σ = 0.266 nm, reported by Li et al.^51^ from fitting to the HFE of Gd^3+^. The fact that a difference <5% is obtained for Gd^3+^ σ after fitting data from different complexes (aqua complex for Li et al., [Gd(DOTA)]^−^ in the present work) to different experimental observables (HFE for Li et al., τ_m_ for us) reinforces the robustness of these parameters.

From the above, we anticipate that Gd^3+^ LJ parameters tuned for use with the DOTA chelate may be used for other Gd^3+^ complexes of similar coordination chemistry. The fact that similar ligand atoms (roughly equal numbers of amino Ns and carbonyl/carboxyl Os) are involved in metal coordination in many Gd-based MRI contrast agents implies similar extents of charge transfer to the Gd ion, and therefore similar effective charge and polarizability of the latter, in most cases. In turn, this means that significant differences in gadolinium LJ parameters are not expected among these complexes.

We tested this hypothesis by simulating the TSAP isomer of [Gd(DOTA)]^−^. We developed a topology for this isomer employing the MCPB procedure (Figure 3), inserting the Gd^3+^ LJ parameters optimized for its SAP counterpart. The results are presented in Table S15 and Figures S14–S17. As shown in Table S15, the previously characterized differences in structure of the two isomers, namely, the IOD (Figure S14), the CN (Figure S14), the distance between the oxygen and nitrogen planes of the complex (Figure S15), angles Ψ (Figure S16), and ω (Figure S17, for illustration of these angles, please refer to Figure 24 of ref (15)) are very well reproduced by our simulations compared to experimental data.^15,100,113^ The kinetics of exchange of the capping water was also determined, and in agreement with experimental findings, it is considerably faster (τ_m_ = 2.4 ns, Figure S5I) than that observed for the SAP isomer.^100,106,114,115^ In previous simulation works, some authors could not obtain a stable TSAP isomer of [Gd(DOTA)]^−^ in solution.^45^ Although others succeed in modeling the TSAP isomer with the capping water present in the Gd^3+^ inner sphere, either the exchange kinetics of this water molecule was not addressed,^36^ or a very short τ_m_ value was obtained.^37^

Overall, this successful brief characterization of the TSAP isomer of [Gd(DOTA)]^−^, using the MCPB procedure and Gd^3+^ LJ parameters derived for the SAP complex, indicates that these parameters are transferable to similarly structured Gd chelates.

## 4 Conclusions

In the present work, we tested different strategies to determine the best protocol for the parametrization of Ln^3+^ complexes for use in classical molecular dynamics. The MCPB procedure revealed superior performance, compared to the heuristic approach based on the work of other authors.^35−37^ The less laborious protocol using the MCPB.py tool makes it a very convenient way to parametrize this kind of complexes using the bonded model. The latter is particularly convenient in the study of metal chelates as a whole, interacting with biological components like proteins, membranes, or nucleic acids. However, this model does not allow the study of the intramolecular dynamics of a lanthanide complex, like conformational changes, and for this purpose other models should be used, such as the 12–6–4 nonbonded model. Our future goal is to simulate these complexes with biomolecules, hence the choice for the bonded model. The best parametrization obtained in this work was TH (with σ = 0.277 nm and ε = 0.244 kJ/mol for Gd^3+^), which could reproduce the structural properties with low RMSD, describe bonds, angles, and improper dihedrals correctly and predict IOD with excellent agreement with experiment. Most importantly, the τ_m_ value obtained for this topology was remarkably accurate. The latter parameter had never previously been estimated by the registration of events of water exchange such as done here, with remarkable accuracy contrasting with the previous poor estimation from PMF calculation.^37^ With this topology, we were able to confirm the dissociative mechanism involved in the inner-sphere water exchange. Furthermore, the dynamics involving the first hydration layer, the intermediate region, and the outer hydration layer were fully investigated. This work clearly shows that the former LJ parameters for the free ion with the simple 12–6 LJ potential were not able to capture the dynamics involving the inner-sphere water exchanging with bulk waters, which led us to optimize the parameters for the Gd^3+^ ion while in the chelated form. Our parameters, developed for the predominant SAP isomer of [Gd(DOTA)]^−^, clearly show superior performance for the chelated form compared to those obtained by other authors,^36,37,39,46,51^ and can be used for similar Gd-based chelates. This was demonstrated by the simulation of the TSAP isomer of [Gd(DOTA)]^−^, reproducing its distinct structural properties and faster water exchange kinetics. The methodology followed in this work may easily be applied to the characterization of all Ln^3+^ ions and their complexes. It is of particular relevance the characterization of a series of Gd^3+^ chelates that lead to different effects on the relaxivity of water protons, and this will be addressed by us in the near future. The methodology developed in this work will allow characterizing the dynamics of the interaction of water molecules with the different complexes, allowing a fine-tuning of their structural properties and the design of new contrast agents with optimized properties.

## Supporting Information Available

Bonded model, parameters conversion to GROMOS 54A7, and the information to fix a bug in the MCPB.py tool within Ambertools 19, and additional Figures and Tables for the SAP and TSAP isomers (PDF)
Complete topology files, compatible with GROMACS, for TD, TH, and TI of the SAP isomer, as well as the equivalent of TH for the TSAP one (ZIP)
