# Supplementary material for: Modeling Gd3+ Complexes for Molecular Dynamics Simulations: Toward a Rational Optimization of MRI Contrast Agents
Source: Inorg Chem. 2022 Jul 18;61(30):11837–58. doi: 10.1021/acs.inorgchem.2c01597 (PMC9775472; doi:10.1021/acs.inorgchem.2c01597)
Supplement: Supplementary file 1 — ic2c01597_si_001.pdf [file ic2c01597_si_001.pdf]

Supporting Information for

Modeling Gd^{3+} Complexes for Molecular Dynamics simulations - Towards a Rational Optimization of MRI Contrast Agents

Alexandre C. Oliveira[a,b], Hugo A. L. Filipe[a,c], João P. Prates Ramalho[d], Armindo Salvador[a,e,f], Carlos F. G. C. Geraldes[a,g,h], Maria João Moreno[a,b*], Luís M. S. Loura[a,i*]

[a] *Coimbra Chemistry Center - Institute of Molecular Sciences (CQC-IMS), University of Coimbra, 3004-535 Coimbra, Portugal*

[b] *Department of Chemistry, University of Coimbra, 3004-535 Coimbra, Portugal*

[c] *CPIRN-IPG—Center of Potential and Innovation of Natural Resources, Polytechnic Institute of Guarda, 6300-559 Guarda, Portugal*

[d] *Hercules Laboratory, LAQV, REQUIMTE, Department of Chemistry, School of Science and Technology, University of Évora, 7000-671 Evora, Portugal*

[e] *CNC—Center for Neuroscience and Cell Biology, University of Coimbra, P-3004-517 Coimbra, Portugal*

[f] *Institute for Interdisciplinary Research - University of Coimbra, Casa Costa Alemão- Polo II, Rua D. Francisco de Lemos, 3030-789 Coimbra, Portugal*

[g] *Department of Life Sciences, University of Coimbra, Calçada Martim de Freitas, 3000-393 Coimbra, Portugal*

[h] *CIBIT/ICNAS - Instituto de Ciências Nucleares Aplicadas à Saúde, Pólo das Ciências da Saúde, Azinhaga de Santa Comba, 3000-548 Coimbra, Portugal*

[i] *Faculty of Pharmacy, University of Coimbra, 3000-548 Coimbra, Portugal*

Keywords: Molecular Dynamics simulations, Lanthanide complexes, $[Gd(DOTA)]^-$, MRI contrast agents, Parameterization, Lennard-Jones parameters, Water residence lifetime, Relaxivity

Table of contents

Contents **Page numbers**

Appendix S.I.1 The bonded model S-2

Appendix S.I.2 Parameter conversion to GROMOS 54A7 S-4

Appendix S.I.3 Bug fixing in the MCPB.py program from Ambertools 19 S-5

Additional Figures and Tables S-6

References S-25

Appendix S.I.1 The bonded model

The parametrization of [Gd(DOTA)]$^-$ was based on the bonded model[1] using the following general potential function within the CGenFF, GAFF and GROMOS force field with the contribution of bonded and non-bonded potential:[2]

$$V(r_{1j}) = V_{bonded} + V_{non-bonded} \quad (1)$$

$$V_{non-bonded} = V_{Van\,der\,Waals} + V_{electrostatic} \quad (2)$$

$$V_{non-bonded}(r_{ij}) = \sum_{i,j\neq i}\left\{\left(\frac{q_i q_j}{4\pi\varepsilon_0 r_{i,j}}\right) + 4\varepsilon_{i,j}\left[\left(\frac{\sigma_{i,j}}{r_{i,j}}\right)^{12} - \left(\frac{\sigma_{i,j}}{r_{i,j}}\right)^{6}\right]\right\} \quad (3)$$

The bonded parameters potential can adopt different potential functions specific to each force field. All the force fields follow the general potential,

$$V_{bonded} = V_{bond-stretch} + V_{angle-bend} + V_{proper-dihedral} + V_{improper-dihedral} \quad (4)$$

with the AMBER force field adopting:

$$V_{bonded}(r_{ijkl}) = \sum_{all-bonds}\frac{1}{2}k_{ij}^{b}(r_{ij} - r_{ij,eq})^2 + \sum_{all-angles}\frac{1}{2}k_{ijk}^{\theta}(\theta_{ijk} - \theta_{ijk,eq}^{0})^2 + \sum_{all-torsions}k_{\phi}(1+\cos(n\phi - \phi_s)) + \sum_{all-improper\ torsions}k_{\xi}(1+\cos(n\xi - \xi_s)) \quad (5)$$

For the CHARMM force field, a different improper dihedral function and the Urey-Bradley (UB) potential for the bond-angle are used:

$$V_{bonded}(r_{ijkl}) = \sum_{all-bonds}\frac{1}{2}k_{ij}^{b}(r_{ij} - r_{ij,eq})^2 + \sum_{all-angles}\frac{1}{2}k_{ijk}^{\theta}(\theta_{ijk} - \theta_{ijk,eq}^{0})^2 + \frac{1}{2}k_{ijk}^{UB}(r_{ik} - r_{ik,eq}^{0})^2 + \sum_{all-torsions}k_{\phi}(1+\cos(n\phi - \phi_s)) + \sum_{all-improper\ torsions}\frac{1}{2}k_{\xi}(\xi - \xi_0)^2 \quad (6)$$

Finally, the GROMOS force field uses fourth power potential for description of the bonds and cosine-based angle potentials:

$$V_{bonded}\left(r_{ijkl}\right) = \sum_{all-bonds} \frac{1}{4} k_{ij}^{b}\left(r_{ij}^{2} - b_{ij}^{2}\right)^{2} + \sum_{all-angles} \frac{1}{2} k_{ijk}^{\theta}\left(\cos\left(\theta_{ijk}\right) - \cos\,(\theta_{ijk}^{0})\right)^{2} + \sum_{all-torsions} k_{\phi}((1 + \cos(n\phi - \phi_{s})) + \sum_{all-improper\ torsions} \frac{1}{2} k_{\xi}(\xi - \xi_{0})^{2} \quad (7)$$

Appendix S.I.2 Parameter conversion to GROMOS 54A7[2]

The bonds in the GAFF force field use a harmonic potential, whereas the GROMOS 54A7 force field uses a fourth power potential, and the conversion of the force constant was obtained using the following equation:

$$2k^{b}b_{ij}^{2} = k^{b,harm} \quad (8)$$

where $k^{b,harm}$ (kJ mol^{-1} nm^{-2}) is the force constant used in the GAFF force field, k^{b} (kJ mol^{-1} nm^{-4}) is the force constant used in the GROMOS 54A7 force field and b_{ij} is the equilibrium bond length (nm). For the bond angles, the GAFF force field uses a harmonic angle potential while the GROMOS 54A7 force field uses a cosine-based angle potential, and the conversion of the force constants was obtained using the following equation:

$$k^{\theta} sin^{2}\left({\theta_{ijk}}^{0}\right) = k^{\theta,harm} \quad (9)$$

where $k^{\theta,harm}$ (kJ mol^{-1} rad^{-2}) is the force constant used in the GAFF force field, k^{θ} (kJ mol^{-1}) is the force constant used in the GROMOS 54A7 force field, and ${\theta_{ijk}}^{0}$ is the equilibrium bond angle in degrees.

The conversion of the non-bonded parameters, the σ_i and ε_i, obtained from the MCPB procedure to $C_i^{(6)}$ and $C_i^{(12)}$ used in the GROMOS 54A7 was carried out according to:

$$C_i^{(6)} = 4\varepsilon_i\sigma_i^{6} \quad [\text{kJ mol}^{-1}\text{nm}^{6}]$$
$$C_i^{(12)} = 4\varepsilon_i\sigma_i^{12} \quad [\text{kJ mol}^{-1}\text{nm}^{12}] \quad (10)$$

The missing pair-types in the topology, namely between Gd^{3+} and the non-coordinated oxygen, were obtained using the combination rules:

$$C_{ij}^{(6)} = (C_i^{(6)}C_j^{(6)})^{1/2}$$
$$C_{ij}^{(12)} = (C_i^{(12)}C_j^{(12)})^{1/2} \quad (11)$$

Appendix S.I.3 Bug fixing in the MCPB.py program from Ambertools 19

After the correct installation of Ambertools 19, edit the file:

```
$AMBERHOME/AmberTools/src/pymsmt/pymsmt/mol/element.py
```

In the line 77 of the code file, change:

```
('GD', 'GD') : ('GD', 64, 157.25),
```

To:

```
('GD', 'GD') : ('Gd', 64, 157.25),
```

Run the following command in the terminal:

```
cd $AMBERHOME/AmberTools/src/pymsmt/ && make install
```

Additional Figures and Tables

Table S1. Parameters from literature that were used in this work with some modifications.

	**Henriques 1999**[3]		**Henriques 2003**[4]		**Dimelow 2007**[5]		
	**Force Field**	CHARMM 22	**Force Field**	CHARMM 22		**Force Field**	AMBER 99
	**Atomic Charge method**	RESP charges determined by *in vacuo* HF calculation with ECP53MWB[6,7] for the Gd^{3+} and 6-31G* for the rest of the atoms	**Atomic Charge method**	RESP charges determined by *in vacuo* HF calculation with ECP53MWB[6,7] for the Gd^{3+} and 6-31G* for the rest of the atoms		**Atomic Charge method**	charges determined by Merz-Kollman method by *in vacuo* DFT calculation using the functional B3LYP/6-311G**
	**Non-bonded parameters**		**Non-bonded parameters**			**Non-bonded parameters**	
**Atom type**	$R_{min}/2$ (Å)	E_{min} (kcal/mol)	$R_{min}/2$ (Å)	E_{min} (kcal mol^{-1})	**atom type**	6–12 radius (σ) (Å)	6–12 energy (ε) (kcal mol^{-1})
Gd3+	1.69	-0.06	1.69	-0.06	Gd	1.69	0.06
	**Bonding parameters**		**Bonding parameters**			**Bonding parameters**	
**Bonds**	Length (Å)	Force Constant (kcal mol^{-1} Å)	Length (Å)	Force Constant (kcal mol^{-1} Å)	**Bonds**	Length (Å)	Force Constant (kcal mol $^{-1}$ Å)
GD3-NT	2.665	95	2.665	190	Gd-NT	2.665	190
GD3-OE	2.365	105	2.365	260	Gd-OS1 and Gd-OS2	2.365	260
	**Angles parameters**		**Angles parameters**			**Angles parameters**	
**Angles**	Angle (deg)	Force Constant (kcal mol^{-1} rad^{-2})	Angle (deg)	Force Constant (kcal mol^{-1} rad^{-2})	**Angles**	Angle (deg)	Force Constant (kcal mol^{-1} rad^{-2})
GD3-NT-CT	111.21	47	111.21	47	Gd–OS1–C and Gd–OS2–C	124.3	95.2
GD3-OE-C	122.38	95.2	124.85	95.2	NT-GD3-NT	80	10
NT-GD3-NT	100	30	80	10	NT-GD3-OS1 and NT-GD3-OS2	94	15
NT-GD3-OE	100	30	94	15	OS1–Gd–OS1and OS2–Gd–OS2	148	10
OE-GD3-OE	100	30	105	10	OS1–Gd–OS2	90	100

Note: All dihedral parameters involving the Gd^{3+} ion were set to zero

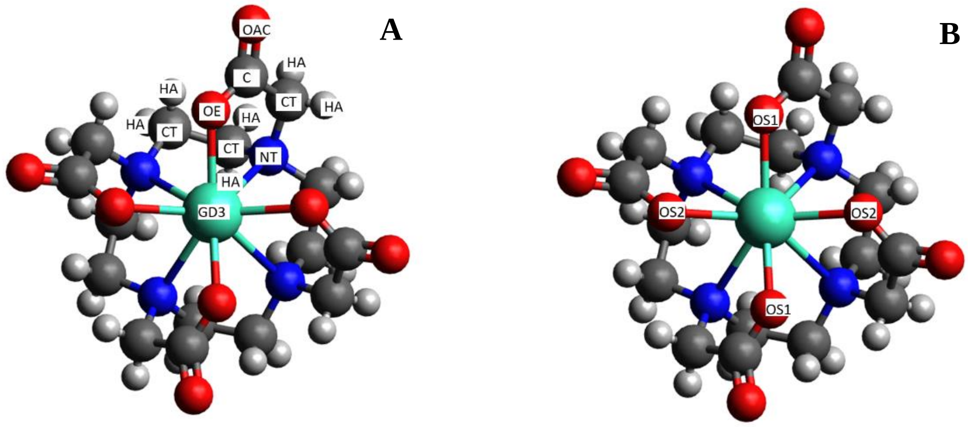

Figure S1. [Gd(DOTA)]$^-$ **s**tructure and atom types adapted from Henriques[3,4] (**A**) and Dimelow[5] (**B**).

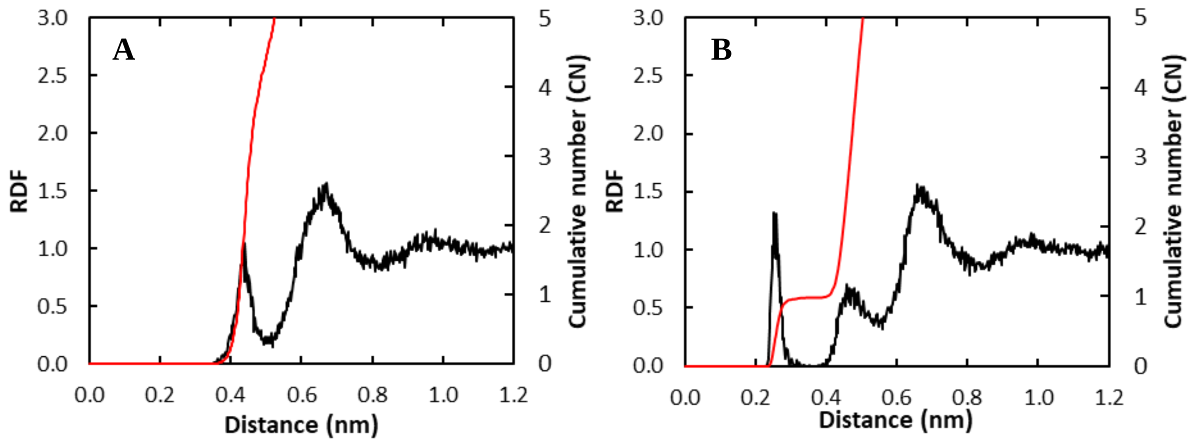

Figure S2. Gd^{3+}-O_{water} RDFs (black) and cumulative number RDFs (red line) for the topology of [Gd(DOTA)]$^-$ using the MK charge model, showing that the inner-sphere water is not present (**A),** and using the RESP charges model, showing the presence of the inner-sphere water (**B)**.

Table S2. Adjustment of the force constant for the bond angle Gd-O-C in the heuristic procedure. All angles are in degree units.

	**k=95.2kcal/mol (398.3 kJ/mol)**				**k=97.5 kcal/mol (407.9 kJ/mol)**				**k=100 kcal/mol (418.4 kJ/mol)**			
**Average Angle (MD)**	114.12	114.20	114.59	114.20	114.47	114.47	114.17	114.64	114.54	114.66	114.85	114.83
**Standard deviation**	4.15	4.11	4.23	4.18	4.10	4.26	3.94	4.20	4.18	4.02	4.24	4.17
**Angle (x-ray crystallography)**	124.83	123.13	125.43	124.97	124.83	123.13	125.43	124.97	124.83	123.13	125.43	124.97
**Topology indexes (Table S4 and Fig. S3)**	17 5 53	19 6 53	21 7 53	23 8 53	17 5 53	19 6 53	21 7 53	23 8 53	17 5 53	19 6 53	21 7 53	23 8 53
**Difference**	10.71	8.93	10.84	10.77	10.35	8.66	11.26	10.33	10.29	8.47	10.58	10.14

	**k=102.5 kcal/mol (428.9 kJ/mol)**				**k=105 kcal/mol (439.3 kJ/mol)**				**k=107.5 kcal/mol (449.8 kJ/mol)**			
**Average Angle (MD)**	114.86	115.02	114.91	114.81	115.06	115.33	114.84	114.83	115.45	115.28	115.00	114.94
**Standard deviation**	4.14	4.02	4.15	4.04	4.03	3.99	3.99	4.00	3.89	3.96	3.95	4.12
**Angle (x-ray crystallography)**	124.83	123.13	125.43	124.97	124.83	123.13	125.43	124.97	124.83	123.13	125.43	124.97
**Topology indexes (Table S4 and Fig. S3)**	17 5 53	19 6 53	21 7 53	23 8 53	17 5 53	19 6 53	21 7 53	23 8 53	17 5 53	19 6 53	21 7 53	23 8 53
**Difference**	9.96	8.12	10.52	10.16	9.77	7.80	10.59	10.14	9.38	7.86	10.43	10.03

	**k=110 kcal/mol (460.2 kJ/mol)**				**k=112.5 kcal/mol (470.7 kJ/mol)**				**k=115 kcal/mol (481.2 kJ/mol)**			
**Average Angle (MD)**	115.43	115.44	115.05	115.46	115.53	115.53	115.58	115.55	115.74	115.87	115.71	115.52
**Standard deviation**	3.86	3.99	3.95	3.83	3.96	3.86	3.79	3.97	4.08	3.71	3.83	3.80
**Angle (x-ray crystallography)**	124.83	123.13	125.43	124.97	124.83	123.13	125.43	124.97	124.83	123.13	125.43	124.97
**Topology indexes (Table S4 and Fig. S3)**	17 5 53	19 6 53	21 7 53	23 8 53	17 5 53	19 6 53	21 7 53	23 8 53	17 5 53	19 6 53	21 7 53	23 8 53
**Difference**	9.40	7.69	10.38	9.51	9.29	7.61	9.85	9.42	9.08	7.26	9.72	9.45

Table S3. Second adjustment of the force constant for the bond angle Gd-O-C after adjustment of the non-bonded parameters. All angles are in degree units.

	k=112.5 kcal/mol (470.7 kJ/mol)				k=115 kcal/mol (481.2 kJ/mol)			
**Average Angle (MD)**	115.60	115.25	115.26	114.92	115.65	115.60	115.41	115.29
**Standard deviation**	3.99	3.85	3.68	4.07	3.77	4.00	3.88	3.94
**Angle (x-ray crystallography)**	124.83	123.13	125.43	124.97	124.83	123.13	125.43	124.97
**Topology indexes (Table S4 and Fig. S3)**	17 5 53	19 6 53	21 7 53	23 8 53	17 5 53	19 6 53	21 7 53	23 8 53
**Difference**	9.22	7.88	10.17	10.05	9.17	7.53	10.02	9.68
	**k=117.5 kcal/mol (491.6 kJ/mol)**				**k=120 kcal/mol (502.1 kJ/mol)**			
**Average Angle (MD)**	115.79	115.49	115.83	115.77	115.99	115.78	115.81	115.95
**Standard deviation**	3.79	3.88	3.77	3.71	3.62	3.90	3.78	3.71
**Angle (x-ray crystallography)**	124.83	123.13	125.43	124.97	124.83	123.13	125.43	124.97
**Topology indexes (Table S4 and Fig. S3)**	17 5 53	19 6 53	21 7 53	23 8 53	17 5 53	19 6 53	21 7 53	23 8 53
**Difference**	9.03	7.64	9.60	9.20	8.84	7.35	9.63	9.02

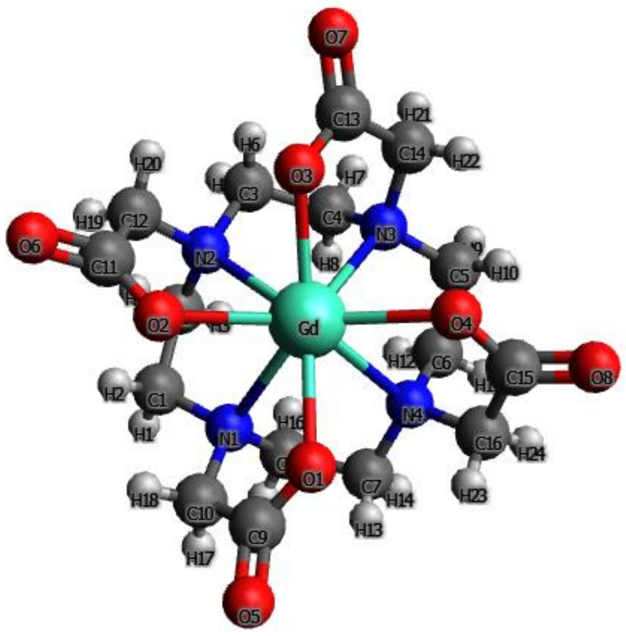

Figure S3. [Gd(DOTA)]$^-$ Structure with the numbering of the atoms used in the heuristic and the MCPB topology.

Table S4. Final topology for the $[Gd(DOTA)]^{-}$ using a heuristic model (TB), using the numbering used in Fig. S3. The remaining parameters of the complex (not shown) are from CGenFF v. 4.1.

	**Atom type**	**Atom**	**Charge**	**Mass**
1	NG301	N1	-0.210	14.007
2	NG301	N2	-0.210	14.007
3	NG301	N3	-0.210	14.007
4	NG301	N4	-0.210	14.007
5	OG2D2	O1	-0.715	15.999
6	OG2D2	O2	-0.715	15.999
7	OG2D2	O3	-0.715	15.999
8	OG2D2	O4	-0.715	15.999
9	CG321	C1	-0.062	12.011
10	CG321	C2	-0.084	12.011
11	CG321	C3	-0.062	12.011
12	CG321	C4	-0.084	12.011
13	CG321	C5	-0.062	12.011
14	CG321	C6	-0.084	12.011
15	CG321	C7	-0.062	12.011
16	CG321	C8	-0.084	12.011
17	CG2O3	C9	0.715	12.011
18	CG321	C10	-0.033	12.011
19	CG2O3	C11	0.715	12.011
20	CG321	C12	-0.033	12.011
21	CG2O3	C13	0.715	12.011
22	CG321	C14	-0.033	12.011
23	CG2O3	C15	0.715	12.011
24	CG321	C16	-0.033	12.011
25	OG2D2	O6	-0.674	15.999
26	OG2D2	O7	-0.674	15.999
27	OG2D2	O8	-0.674	15.999
28	OG2D2	O9	-0.674	15.999
29	HGA2	H1	0.039	1.008
30	HGA2	H2	0.106	1.008
31	HGA2	H3	0.069	1.008
32	HGA2	H4	0.073	1.008
33	HGA2	H5	0.039	1.008
34	HGA2	H6	0.106	1.008
35	HGA2	H7	0.073	1.008
36	HGA2	H8	0.069	1.008
37	HGA2	H9	0.039	1.008
38	HGA2	H10	0.106	1.008
39	HGA2	H11	0.073	1.008
40	HGA2	H12	0.069	1.008
41	HGA2	H13	0.106	1.008
42	HGA2	H14	0.039	1.008
43	HGA2	H15	0.073	1.008
44	HGA2	H16	0.069	1.008
45	HGA2	H17	0.021	1.008
46	HGA2	H18	0.070	1.008
47	HGA2	H19	0.021	1.008
48	HGA2	H20	0.070	1.008
49	HGA2	H21	0.021	1.008
50	HGA2	H22	0.070	1.008
51	HGA2	H23	0.070	1.008
52	HGA2	H24	0.021	1.008
53	GD3	Gd1	1.740	157.25

	**Force Field**	CGenFF v. 4.1
	**Atomic Charge method**	Charges determined by RESP method of the optimized structure *in vacuo* using HF level of theory with the 6-31G* basis function and Dolg *et al.* ECP for the Gd^{3+}.[6,7]
	**non bonded parameters**	
**atom type**	6–12 radius (σ) (nm)	6–12 energy (ε) (kJ mol^{-1})
GD3	0.281266432	0.2892154
	**Bonding parameters**	
**Bonds**	Length (nm)	Force Constant (kJ mol^{-1} nm^{-2})
Gd-NG301	0.2665	79496
Gd-OG2D2	0.2365	108784
	**Angle parameters**	
**Angles**	Angle (deg)	Force Constant/ kcal mol^{-1} rad^{-2} (kJ mol^{-1} rad^{-2})
Gd–O–C	124.575	117.5 (491.620)
N1-Gd-N3 and N2-Gd-N4	104.400	10.0 (41.840)
N1-Gd-N2; N2-Gd-N3; N3-Gd-N4 and N4-Gd-N1	67.900	10.0 (41.840)
O1-Gd-O3 and O2-Gd-O4	144.800	10.0 (41.840)
O1-Gd-O2; O2-Gd-O3; O3-Gd-O4 and O4-Gd-O1	84.775	100.0 (418.400)
N1-Gd-O1; N2-Gd-O2; N3-Gd-O3 and N4-Gd-O4	66.200	15.0 (62.760)
N1-Gd-O2; N2-Gd-O3; N3-Gd-O4 and N4-Gd-O1	73.475	15.0 (62.760)
N1-Gd-O4; N2-Gd-O1; N3-Gd-O2 and N4-Gd-O3	130.875	15.0 (62.760)
N1-Gd-O3; N2-Gd-O4; N3-Gd-O1 and N4-Gd-O2	140.725	15.0 (62.760)
Gd-N1-C10; Gd-N2-C12; Gd-N3-C14 and Gd-N4-C16	106.250	47.0 (196.648)
Gd-N1-C8; Gd-N2-C2; Gd-N3-C4 and Gd-N4-C6	111.200	47.0 (196.648)
Gd-N1-C1; Gd-N2-C3; Gd-N3-C5; Gd-N4-C7	110.875	47.0 (196.648)
	**Dihedrals**	
	All dihedral parameters involving the Gd^{3+} ion were set to zero	

Table S5. Adjustment of the force constant for the O-Gd-O angle for the heuristic topology using the LJ parameters developed by Li *et al.* to reproduce HFE[8]. All angles are in degree units.

	k=10 kcal/mol (41.8 kJ/mol)		k= 12.5 kcal/mol (52.3 kJ/mol)		k= 15 kcal/mol (62.8 kJ/mol)	
**Average Angle (MD)**	153.79	154.10	153.57	153.46	153.40	153.40
**Standard deviation**	4.01	4.06	3.92	4.13	3.98	3.95
**Angle (x-ray crystallography)**	145.91	143.71	145.91	143.71	145.91	143.71
**Topology indexes (Table S4 and Fig. S3)**	5 53 7	6 53 8	5 53 7	6 53 8	5 53 7	6 53 8
**Difference**	7.89	10.39	7.66	9.75	7.49	9.69

	k= 17.5 kcal/mol (73.2 kJ/mol)		k= 20 kcal/mol (83.7 kJ/mol)	
**Average Angle (MD)**	153.18	153.23	152.76	152.95
**Standard deviation**	3.96	4.01	3.89	3.97
**Angle (x-ray crystallography)**	145.91	143.71	145.91	143.71
**Topology indexes (Table S4 and Fig. S3)**	5 53 7	6 53 8	5 53 7	6 53 8
**Difference**	7.27	9.52	6.86	9.23

Table S6. Adjustment of the force constant for the Gd-O-C angle for the heuristic topology using the LJ parameters developed by Li *et al.* to reproduce HFE[8]. All angles are in degree units.

	k=117.5 kcal/mol (491.6 kJ/mol)				k=120.0 kcal/mol (502.1 kJ/mol)			
**Average Angle (MD)**	115.54	115.37	115.43	115.30	115.74	115.58	115.51	115.47
**Standard deviation**	3.73	3.87	3.85	3.72	3.73	3.89	3.83	3.65
**Angle (x-ray crystallography)**	124.83	123.13	125.43	124.97	124.83	123.13	125.43	124.97
**Topology indexes (Table S4 and Fig. S3)**	17 5 53	19 6 53	21 7 53	23 8 53	17 5 53	19 6 53	21 7 53	23 8 53
**Difference**	9.28	7.77	10.00	9.67	9.09	7.55	9.92	9.50

Table S7. Changes in the final topology for $[Gd(DOTA)]^-$ from the table S4 using a heuristic model (TD). The full topology is available in the Supporting Information.

	**non bonded parameters**	
**atom type**	6–12 radius (σ) (nm)	6–12 energy (ε) (kJ mol^{-1})
GD3	0.2513	0.07300
	**Bonding parameters**	
	**Angles parameters**	
**Angles**	Angle (deg)	Force Constant/ kcal mol^{-1} rad^{-2} (kJ mol^{-1} rad^{-2})
Gd–O–C	124.6	120 (502.1)
O1-Gd-O3 and O2-Gd-O4	144.8	12.5 (52.3)

Table S8. Final topology for the $[Gd(DOTA)]^-$ using the MCPB procedure with the non-bonded parameters adjusted to the experimental IOD and τ_m, topology TF and TH, respectively, using the numbering used in the Figure S3. The remaining parameters of the complex (not shown) are from GAFF force field. The full topology is available in the Supporting Information.

	**Atom type**	**Atom**	**Charge**	**Mass**
1	M1	GD	2.024	157.250
2	Y1	N1	-0.119	14.010
3	Y2	N2	-0.119	14.010
4	Y3	N3	-0.119	14.010
5	Y4	N4	-0.119	14.010
6	Y5	O1	-0.829	16.000
7	Y6	O2	-0.829	16.000
8	Y7	O3	-0.829	16.000
9	Y8	O4	-0.829	16.000
10	c3	C1	-0.042	12.010
11	c3	C2	-0.232	12.010
12	c3	C3	-0.042	12.010
13	c3	C4	-0.232	12.010
14	c3	C5	-0.042	12.010
15	c3	C6	-0.232	12.010
16	c3	C7	-0.042	12.010
17	c3	C8	-0.232	12.010
18	c	C9	0.823	12.010
19	c3	C10	-0.305	12.010
20	c	C11	0.823	12.010
21	c3	C12	-0.305	12.010
22	c	C13	0.823	12.010
23	c3	C14	-0.305	12.010
24	c	C15	0.823	12.010
25	c3	C16	-0.305	12.010
26	o	O5	-0.618	16.000
27	o	O6	-0.618	16.000
28	o	O7	-0.618	16.000
29	o	O8	-0.618	16.000
30	h1	H1	0.055	1.008
31	h1	H2	0.101	1.008
32	h1	H3	0.095	1.008
33	h1	H4	0.107	1.008
34	h1	H5	0.055	1.008
35	h1	H6	0.101	1.008
36	h1	H7	0.107	1.008
37	h1	H8	0.095	1.008
38	h1	H9	0.055	1.008
39	h1	H10	0.101	1.008
40	h1	H11	0.107	1.008
41	h1	H12	0.095	1.008
42	h1	H13	0.101	1.008
43	h1	H14	0.055	1.008
44	h1	H15	0.107	1.008
45	h1	H16	0.095	1.008
46	h1	H17	0.072	1.008
47	h1	H18	0.136	1.008
48	h1	H19	0.072	1.008
49	h1	H20	0.136	1.008
50	h1	H21	0.072	1.008
51	h1	H22	0.136	1.008
52	h1	H23	0.136	1.008
53	h1	H24	0.072	1.008

	**Force Field**	GAFF
	**Atomic Charge method**	Charges determined by RESP method of the optimized structure *in vacuo* using DFT level of theory with B3LYP functional and the 6-31G* basis function and Dolg *et al.* ECP for the Gd^{3+}.[6,7]
	**Non-bonded parameters**	
**Atom type**	6–12 radius (σ) (nm)	6–12 energy (ε) (kJ mol^{-1})
M1 (Gd) IOD (TF)	0.285771	0.340010
M1 (Gd) τ_m (TH)	0.276863	0.244429
	**Bonding parameters**	
**Bonds**	Length (nm)	Force Constant (kJ mol^{-1} nm^{-2})
GD-N	0.274	4267.70
GD-O	0.233	45857.00
	**Angle parameters**	
**Angles**	Angle (deg)	Force Constant/ kJ mol^{-1} rad^{-2}
GD-N-C	107.97	752.20
GD-O-C	126.85	461.75
N1-GD-N3; N2-GD-N4	102.30	200.58
N1-GD-N2; N2-GD-N3; N3-GD-N4; N4-GD-N1	66.83	348.61
O1-GD-O3; O2-Gd-O4	144.40	642.83
O1-GD-O2; O2-GD-O3; O3-GD-O4; O4-GD-O1	84.64	465.43
N1-GD-O1; N2-GD-O2; N3-GD-O3; N4-GD-O4	67.12	535.13
N1-GD-O2; N2-GD-O3; N3-GD-O4; N4-GD-O1	74.36	289.62
N1-GD-O4; N2-GD-O1; N3-GD-O2; N4-GD-O3	130.78	498.57
N1-GD-O3; N2-GD-O4; N3-GD-O1; N4-GD-O2	140.56	313.13
	**Dihedrals**	
	All dihedrals parameters involving the Gd^{3+} ion were set to zero	

Table S9. Adjustment of the force constant for the O-C-Gd angle for the United-atom procedure. All angles are in degree units.

	k= 721.1 kJ/mol				k= 750.0 kJ/mol				k=800.0 kJ/mol			
**Average Angle (MD)**	110.58	110.87	111.00	110.95	111.30	111.41	111.33	111.46	112.05	112.01	112.12	112.55
**Standard deviation**	3.26	3.39	3.12	3.51	3.07	3.37	3.07	3.56	3.21	3.32	3.24	3.46
**Angle (x-ray crystallography)**	124.83	123.13	125.43	124.97	124.83	123.13	125.43	124.97	124.83	123.13	125.43	124.97
**Topology indexes (Table S10 and Fig. S4)**	2 3 29	10 12 29	17 18 29	26 27 29	2 3 29	10 12 29	17 18 29	26 27 29	2 3 29	10 12 29	17 18 29	26 27 29
**Difference**	14.24	12.26	14.44	14.02	13.52	11.73	14.11	13.51	12.77	11.13	13.31	12.42

	k=850.0 kJ/mol				k=900.0 kJ/mol				k=950.0 kJ/mol			
**Average Angle (MD)**	112.80	112.78	112.74	113.07	113.33	113.55	113.55	113.72	113.92	114.17	114.03	114.52
**Standard deviation**	2.91	3.12	3.21	3.37	2.96	3.08	3.15	3.25	2.99	3.01	3.11	3.29
**Angle (x-ray crystallography)**	124.83	123.13	125.43	124.97	124.83	123.13	125.43	124.97	124.83	123.13	125.43	124.97
**Topology indexes (Table S10 and Fig. S4)**	2 3 29	10 12 29	17 18 29	26 27 29	2 3 29	10 12 29	17 18 29	26 27 29	2 3 29	10 12 29	17 18 29	26 27 29
**Difference**	12.03	10.35	12.69	11.90	11.49	9.58	11.88	11.25	10.90	8.96	11.40	10.45

	k=1000.0 kJ/mol				k=1050.0 kJ/mol				k=1100.0 kJ/mol			
**Average Angle (MD)**	114.27	114.45	114.60	115.18	114.87	115.01	115.12	115.53	115.48	115.38	115.49	115.88
**Standard deviation**	2.92	2.93	3.04	3.21	2.91	3.01	2.98	3.08	2.89	2.88	2.80	3.11
**Angle (x-ray crystallography)**	124.83	123.13	125.43	124.97	124.83	123.13	125.43	124.97	124.83	123.13	125.43	124.97
**Topology indexes (Table S10 and Fig. S4)**	2 3 29	10 12 29	17 18 29	26 27 29	2 3 29	10 12 29	17 18 29	26 27 29	2 3 29	10 12 29	17 18 29	26 27 29
**Difference**	10.55	8.68	10.83	9.79	9.96	8.13	10.32	9.44	9.34	7.75	9.94	9.09

	k=1150.0 kJ/mol				k=1200.0 kJ/mol			
**Average Angle (MD)**	115.76	115.95	115.93	116.37	116.12	116.26	116.29	117.13
**Standard deviation**	2.73	2.91	2.87	2.95	2.83	2.71	2.86	2.91
**Angle (x-ray crystallography)**	124.83	123.13	125.43	124.97	124.83	123.13	125.43	124.97
**Topology indexes (Table S10 and Fig. S4)**	2 3 29	10 12 29	17 18 29	26 27 29	2 3 29	10 12 29	17 18 29	26 27 29
**Difference**	9.07	7.18	9.50	8.60	8.70	6.87	9.14	7.84

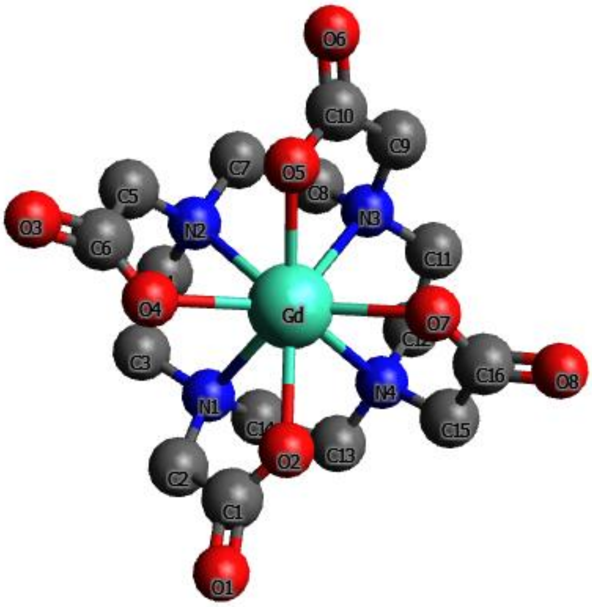

Figure S4. [Gd(DOTA)]$^{-}$ structure with the numbering of the atoms used in the United-atom procedure (topology TI).

Table S10. Final topology for the $[Gd(DOTA)]^-$ obtained in the United-atom procedure, topology TI, using the numbering used in the Figure S4. The remaining parameters of the complex (not shown) are from the GROMOS 54A7 force field. The full topology is available in the Supporting Information.

	**Atom type**	**Atom**	**Charge**	**Mass**
1	OM	O	-0.628	15.999
2	CPos	C	0.873	12.011
3	OM	O	-0.855	15.999
4	CH2	C	-0.135	14.027
5	NTer	N	-0.114	14.007
6	CH2	C	0.149	14.027
7	CH2	C	-0.063	14.027
8	NTer	N	-0.114	14.007
9	CH2	C	-0.135	14.027
10	CPos	C	0.873	12.011
11	OM	O	-0.628	15.999
12	OM	O	-0.855	15.999
13	CH2	C	0.149	14.027
14	CH2	C	-0.063	14.027
15	NTer	N	-0.114	14.007
16	CH2	C	-0.135	14.027
17	CPos	C	0.873	12.011
18	OM	O	-0.855	15.999
19	OM	O	-0.628	15.999
20	CH2	C	0.149	14.027
21	CH2	C	-0.063	14.027
22	NTer	N	-0.114	14.007
23	CH2	C	0.149	14.027
24	CH2	C	-0.063	14.027
25	CH2	C	-0.135	14.027
26	CPos	C	0.873	12.011
27	OM	O	-0.855	15.999
28	OM	O	-0.628	15.999
29	GD3	GD	2.092	157.250

	**Force Field**	GROMOS 54A7
	**Atomic Charge method**	Charges determined by MK method of the optimized structure *in vacuo* using DFT level of theory with B3LYP functional and the 6-31G* basis function and Dolg *et al.* ECP for the Gd^{3+}.[6,7]
	**Non-bonded parameters**	
**Atom type**	C_i^6 (kJ mol^{-1}nm^6)	C_i^{12} (kJ mol^{-1} nm^{12})
GD3	4.404×10^{-4}	1.983×10^{-7}
	**Bonding parameters**	
**Bonds**	Length (nm)	Force Constant (kJ mol^{-1} nm^{-4})
GD-N	0.274	2.832×10^{4}
GD-O	0.233	4.216×10^{5}
	**Angle parameters**	
**Angles**	Angle (deg)	Force Constant/ kJ mol^{-1}
GD-N-C	107.97	831.33
GD-O-C	126.85	1100.00
N1-GD-N3; N2-GD-N4	102.30	210.12
N1-GD-N2; N2-GD-N3; N3-GD-N4; N4-GD-N1	66.83	412.46
O2-GD-O5; O4-GD-O7	144.40	1897.00
O2-GD-O4; O4-GD-O5; O5-GD-O7; O7-GD-O2	84.64	469.53
N1-GD-O2; N2-GD-O4; N3-GD-O5; N4-GD-O7	67.12	630.43
N1-GD-O4; N2-GD-O5; N3-GD-O7; N4-GD-O2	74.36	312.32
N1-GD-O7; N2-GD-O2; N3-GD-O4; N4-GD-O5	130.78	869.52
N1-GD-O5; N2-GD-O7; N3-GD-O2; N4-GD-O4	140.56	775.90
	**Dihedrals**	
	All dihedrals parameters involving the Gd^{3+} ion was set to zero	

Table S11. Adjustment of the non-bonded parameters using the 12-6 LJ model to reproduce the experimental IOD for the heuristic and MCPB procedure.

				Heuristic procedure		MCPB procedure	
	$R_{min}/2$ (A)	σ (nm)	ε kJ/mol	IOD (nm)	CN	IOD (nm)	CN
	1.300	0.232	0.021	0.195	9.00	0.198	9.00
	1.350	0.241	0.038	0.204	9.00	0.208	9.00
	1.400	0.249	0.066	0.216	9.00	0.215	9.00
	1.450	0.258	0.106	0.224	9.00	0.224	9.00
	1.500	0.267	0.163	0.235	9.00	0.232	9.00
	1.550	0.276	0.238	0.241	9.00	0.240	9.00
	1.600	0.285	0.332	0.253	9.00	0.250	9.00
	1.650	0.294	0.446	0.264	8.93	0.255	9.00
	1.700	0.303	0.578	0.272	8.90	0.264	9.00
	1.750	0.312	0.728	0.278	8.83	0.271	9.00
	1.800	0.321	0.892	0.283	8.74	0.279	9.00
	1.850	0.330	1.067	0.297	8.68	0.284	9.00
	1.900	0.339	1.251	0.301	8.54	0.292	8.96
**Optimized parameters for the Heuristic procedure**	1.579	0.281	0.289	0.247	9.00		
**Optimized parameters for the MCPB procedure**	1.604	0.286	0.340			0.249	9.00

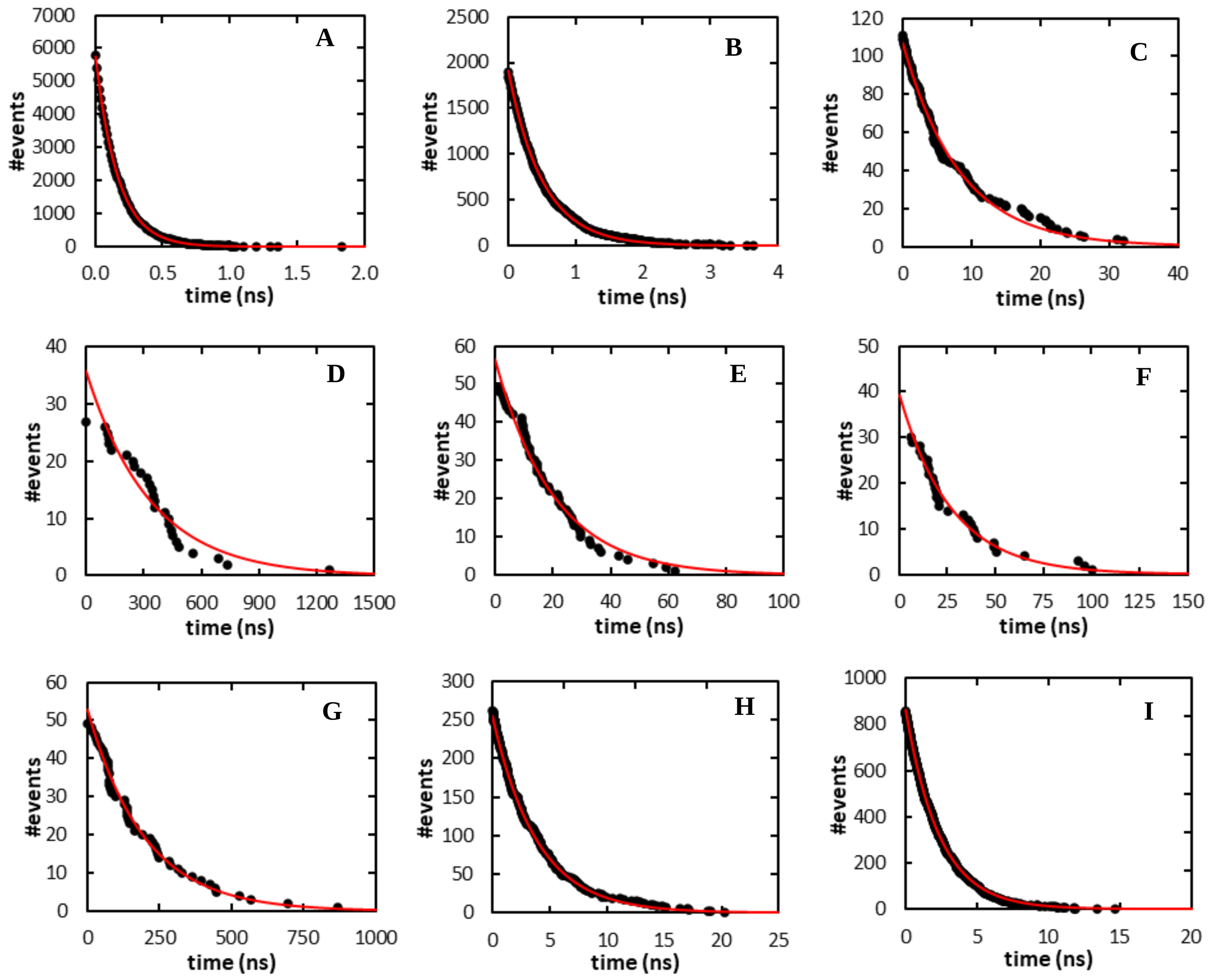

Figure S5. Events of water exchange in the metal center that allow the calculation of the mean residency time (τ_m) of the water in the metal center inner sphere for the topology TA **(A)**, topology TB **(B)**, topology TC **(C)**, topology TD **(D)**, topology TE **(E)**, topology TF **(F)**, topology TH **(G)**, topology TI **(H)** and for the topology obtained with the procedure and parameters obtained from TH but applied to the TSAP geometry (**I**).

Table S12. Adjustment of the non-bonded parameters using the 12-6 LJ model for the Heuristic and MCPB procedure in order to reproduce τ_m.

	$R_{min}/2$ (Å)	σ (nm)	ε (kJ mol^{-1})	k (ns^{-1})	τ (ns)	# events observed	Simulation time (μs)
**Heuristic procedure**	1.400	0.249	0.0657	2.954×10^{-3}	338.5	12	5
	1.450	0.258	0.1065	1.374×10^{-2}	72.78	74	5
	1.500	0.267	0.1632	1.549×10^{-1}	6.454	774	5
	1.550	0.276	0.2379	8.889×10^{-1}	1.125	879	1
	1.600	0.285	0.3320	3.584×10^{-0}	0.279	3458	1
	1.650	0.294	0.4457	$1.064\times10^{+1}$	0.094	9979	1
	1.700	0.303	0.5782	$2.500\times10^{+1}$	0.040	20256	1
**Optimized parameters**	1.410	0.251	0.0730	2.984×10^{-3}	335.1	27	10
**MCPB procedure**	1.550	0.276	0.2380	2.334×10^{-3}	428.4	15	5
	1.561	0.278	0.2580	6.757×10^{-3}	148.0	68	10
	1.575	0.281	0.2820	8.816×10^{-3}	113.4	21	2
	1.600	0.285	0.3320	1.720×10^{-2}	58.15	41	2
	1.650	0.294	0.4460	1.042×10^{-1}	9.594	106	1
	1.700	0.303	0.5780	4.619×10^{-1}	2.165	464	1
**Optimized parameters**	1.554	0.277	0.2444	5.074×10^{-3}	197.1	49	10

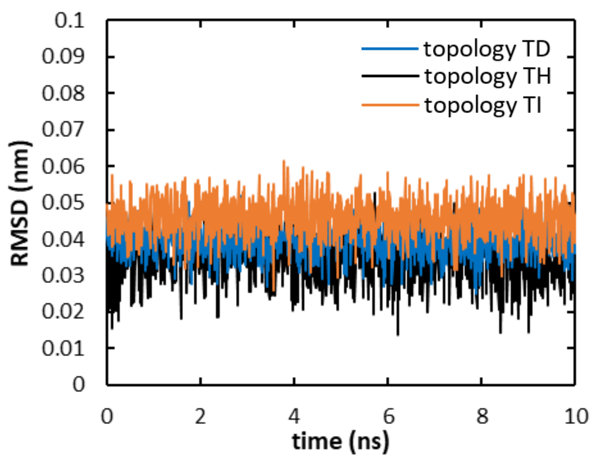

Figure S6. Comparison of the Residual Mean Square Deviation (RMSD) of the $[Gd(DOTA)]^-$ structure obtained using the heuristic procedure (TD), MCPB procedure (TH) and United-atom procedure (TI) with the X-ray crystallographic structure of $[Gd(DOTA)]^-$.

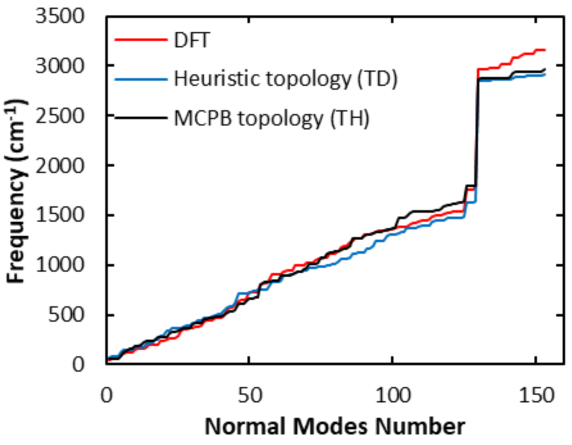

Figure S7. Comparison of the calculated normal modes from Density Functional Theory (DFT) using B3LYP functional with effective core potential (ECP) of Dolg *et al.* for the Gadolinium[6,7] and 6-31G* for the remaining atoms (red line) and from Molecular Mechanics using the heuristic procedure (topology TD, blue line) and MCPB procedure (topology TH, black line).

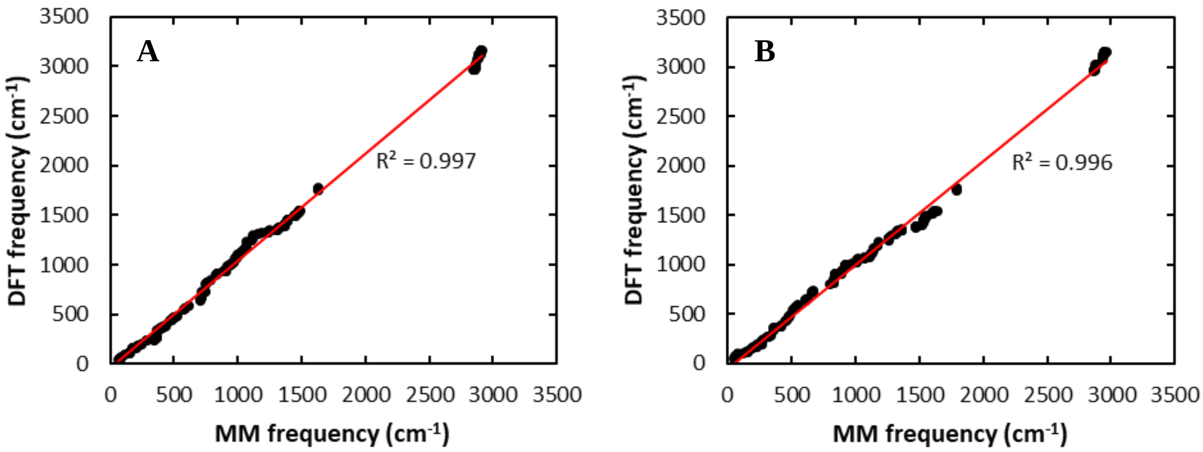

Figure S8. Linear fitting of the DFT and MM calculated normal modes for the heuristic procedure (TD) **(A)** and for the MCPB procedure (TH) **(B)**.

Table S13. Kinetic study of the inner sphere and 1st hydration layer using the topology (TH) varying the non-bonded parameters.

	$R_{min}/2$ (Å)	τ_m (ns)	#events observed Inner sphere	cut-off (inner sphere and 1st hydration layer)	$\tau_{1st\ hydration\ layer}$ (ps)	#number of water molecules in 1st hydration layer
	1.550	428.4	15	0.21-0.33; 0.38-0.53	18.4	6.0
	1.561	148.0	68	0.21-0.33; 0.38-0.53	18.3	6.0
	1.575	113.4	21	0.23-0.34; 0.38-0.54	18.4	6.3
	1.600	58.15	41	0.23-0.34; 0.38-0.54	18.4	6.3
	1.650	9.594	106	0.23-0.34; 0.38-0.54	18.2	6.2
	1.700	2.165	464	0.23-0.34; 0.38-0.54	17.9	6.2
**Optimized parameters**	1.554	197.1	49	0.21-0.33; 0.38-0.53	18.4	6.0

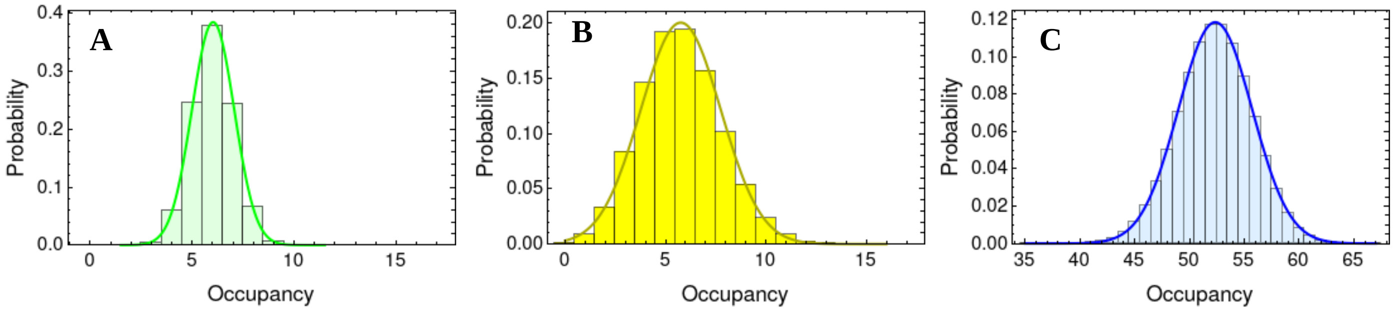

Figure S9. Distribution of the number of water molecules in the various layers of the outer hydration sphere of $[Gd(DOTA)]^-$ using the topology TH, first hydration layer **(A)**, intermediate region **(B)** and outer hydration layer **(C).** The bars correspond to the observed distribution, and the lines are the best fit of a Gaussian-shaped function.

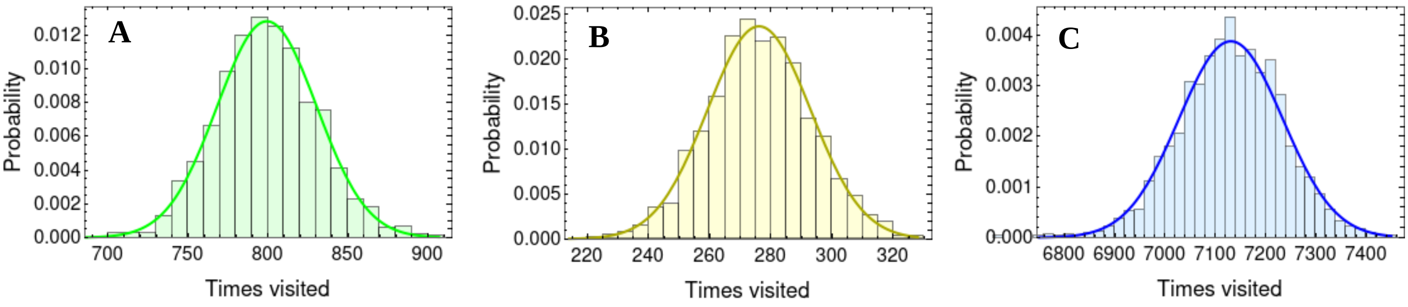

Figure S10. Histogram showing the distribution of visits of water molecules (with a residence of at least two consecutive frames, 20 ps) to the various layers of the outer hydration sphere of $[Gd(DOTA)]^-$ with the topology TH, with on average 799 visits (standard deviation of 275) for the first hydration layer **(A)**, 276 visits (standard deviation of 17) for the intermediate region **(B)** and 7131 visits (standard deviation of 103) for the outer hydration layer **(C)**. The bars correspond to the observed distribution, and the lines are the best fit of a Gaussian-shaped function.

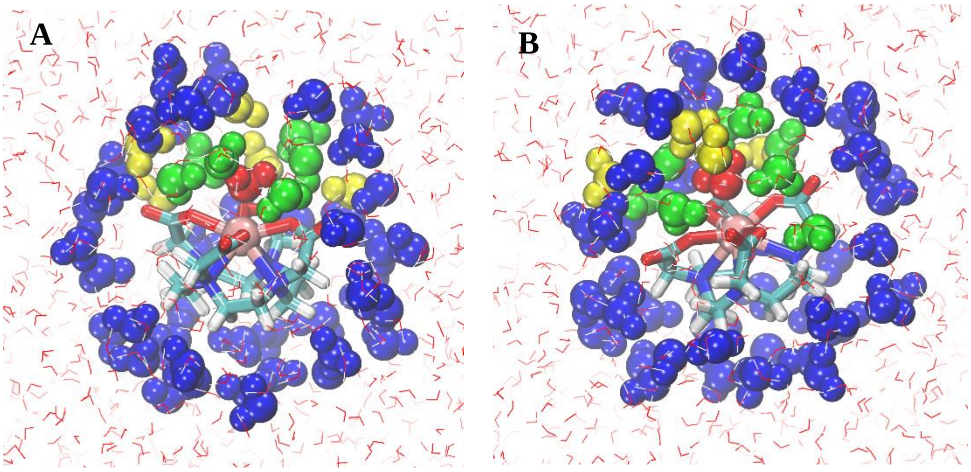

Figure S11. Snapshots of the system at 944700 ps **(A)** and 944800 ps **(B)** showing the organizations of the waters in different layers, the inner sphere water (red), the first hydration layer (green), the intermediate region (yellow) and the outer hydration layer (blue). Some water molecules were removed from this last region.

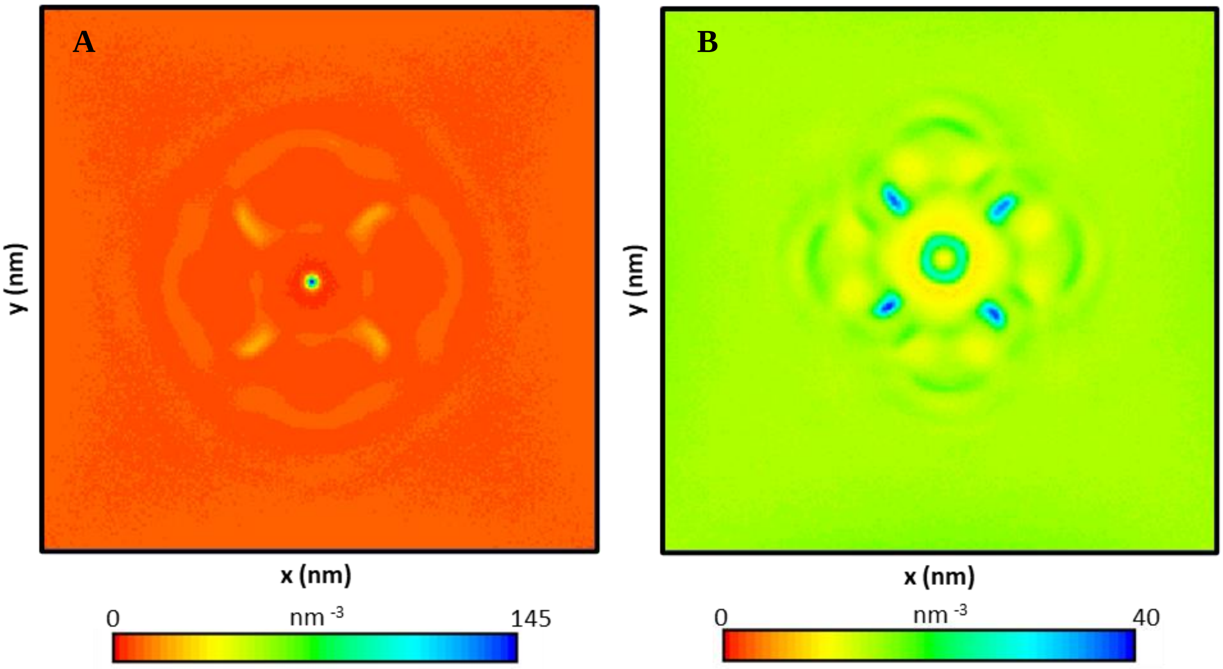

Figure S12. Density map for the oxygen **(A)** and hydrogen **(B)** of the water molecules around $[Gd(DOTA)]^-$ with the topology TH. The complex was centered in middle of the system box with its translation and rotation removed post-simulation. The density map was set in the direction to the normal of the plane xy with z centered in Gd^{3+} averaged inside a distance of 0.8 nm from the hydrophilic part of the complex.

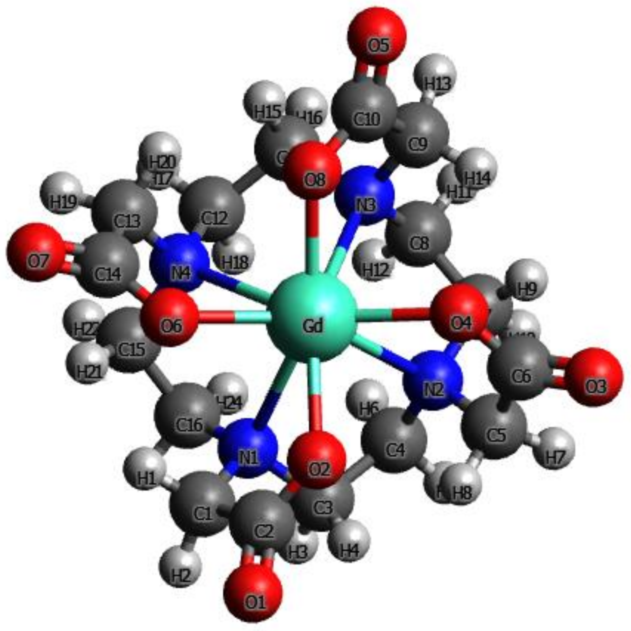

Figure S13. [Gd(DOTA)]$^{-}$ Structure in the TSAP geometry with the numbering of the atoms.

Table S14. Final topology for the [Gd(DOTA)]- in the TSAP geometry using the MCPB procedure with the non-bonded parameters from topology TH, using the numbering used in the Figure S13. The remaining parameters of the complex (not shown) are from GAFF force field. The full topology is available in the Supporting Information.

	**Atom type**	**Atom**	**Charge**	**Mass**
1	M1	GD	1.933	157.250
2	Y1	N1	-0.100	14.010
3	Y2	N2	-0.100	14.010
4	Y3	N3	-0.100	14.010
5	Y4	N4	-0.100	14.010
6	o	O1	-0.590	16.000
7	Y5	O2	-0.785	16.000
8	o	O3	-0.590	16.000
9	Y6	O4	-0.785	16.000
10	o	O5	-0.590	16.000
11	Y7	O6	-0.785	16.000
12	o	O7	-0.590	16.000
13	Y8	O8	-0.785	16.000
14	c3	C1	-0.336	12.010
15	h1	H1	0.160	1.008
16	h1	H2	0.080	1.008
17	c	C2	0.753	12.010
18	c3	C3	-0.096	12.010
19	h1	H3	0.062	1.008
20	h1	H4	0.115	1.008
21	c3	C4	-0.164	12.010
22	h1	H5	0.089	1.008
23	h1	H6	0.080	1.008
24	c3	C5	-0.336	12.010
25	h1	H7	0.080	1.008
26	h1	H8	0.160	1.008
27	c	C6	0.753	12.010
28	c3	C7	-0.096	12.010
29	h1	H9	0.115	1.008
30	h1	H10	0.062	1.008
31	c3	C8	-0.164	12.010
32	h1	H11	0.089	1.008
33	h1	H12	0.080	1.008
34	c3	C9	-0.336	12.010
35	h1	H13	0.080	1.008
36	h1	H14	0.160	1.008
37	c	C10	0.753	12.010
38	c3	C11	-0.096	12.010
39	h1	H15	0.115	1.008
40	h1	H16	0.062	1.008
41	c3	C12	-0.164	12.010
42	h1	H17	0.089	1.008
43	h1	H18	0.080	1.008
44	c3	C13	-0.336	12.010
45	h1	H19	0.080	1.008
46	h1	H20	0.160	1.008
47	c	C14	0.753	12.010
48	c3	C15	-0.096	12.010
49	h1	H21	0.115	1.008
50	h1	H22	0.062	1.008
51	c3	C16	-0.164	12.010
52	h1	H23	0.089	1.008
53	h1	H24	0.080	1.008

	**Force Field**	GAFF
	**Atomic Charge method**	charges determined by RESP method of the optimized structure *in vacuo* using DFT level of theory with B3LYP functional and the 6-31G* basis function and Dolg *et al.* ECP for the Gd^{3+}.[6,7]
	**non bonded parameters**	
**atom type**	6–12 radius (σ) (Å)	6–12 energy (ε) (kcal mol^{-1})
M1 (Gd)	0.276863	0.244429
	**Bonding parameters**	
**Bonds**	Length (nm)	Force Constant (kJ $mol^{-1}nm^{-2}$)
GD-N	0.27711	5104.50
GD-O	0.23316	44016.00
	**Angle parameters**	
**Angles**	Angle (deg)	Force Constant/ kJ $mol^{-1}rad^{-2}$
GD-N-C	107.86	629.11
GD-O-C	125.94	308.53
N1-GD-N3; N2-GD-N4	99.81	460.74
N1-GD-N2; N2-GD-N3; N3-GD-N4; N4-GD-N1	65.49	283.34
O2-GD-O8; O4-GD-O6	141.57	659.90
O2-GD-O4; O4-GD-O8; O8-GD-O6; O6-GD-O2	83.78	468.69
N1-GD-O2; N2-GD-O4; N3-GD-O8; N4-GD-O6	63.96	598.23
N1-GD-O6; N4-GD-O8; N3-GD-O4; N2-GD-O2	84.19	116.57
N1-GD-O4; N4-GD-O2; N3-GD-O6; N2-GD-O8	121.68	419.57
N1-GD-O8; N4-GD-O4; N3-GD-O2; N2-GD-O6	149.64	182.17
	**Dihedrals**	
All diehdral parameters involving the gadolinium ion was set to zero		

Table S15. Analysis results of the $[Gd(DOTA)]^-$ in the square antiprismatic (SAP) and twisted antiprismatic conformation (TSAP). For each form, the IOD, the CN, the distance between the oxygen and nitrogen planes of the complex, and angles Ψ and ω (the opening angle Ψ is formed between the opposite coordinated oxygens and the Gd^{3+} are shown. The twist angle ω is formed between the planar projections of the vectors defined by the Nitrogens to the Gd^{3+} and the vectors defined between the coordinated oxygens of the same acetate arm and the Gd^{3+} ion. For illustration of these angles, refer to Fig. 24 of reference [9]), the kinetics of exchange waters in the inner sphere of the metal ion, and the ratio of those kinetics between the SAP and TSAP conformations. The experimental data available in the literature is presented for comparison. Values inside parentheses are standard deviations.

[Gd(DOTA)]- Conformation	IOD (nm)	CN	Distance between O-N plane	Angle Ψ	Twist angle ω	τ_m (ns)	τ_m(SAP)/ τ_m(TSAP)	
SAP (parametrized, values from MD)	0.240	9.00	0.235 (0.003)	146.79 (1.73)	37.67 (1.13)	197	MD	82
TSAP (parametrized, values from MD)	0.248	9.00	0.247 (0.003)	143.47 (1.71)	24.06 (1.33)	2.4		
Experimental SAP	0.243[a),11]	9.00[a),11]	0.236[9]	148[9]	37.6[9]	360[10] 70[c),12]		
Experimental TSAP	0.250[b),10]	9.00[b),10]	0.252[9]	144[9]	24.5[9]	53[10] 6[d),12]	Exp.	7,[10] 12,[12] ~50[e),13], 44[f),17]
Experimental SAP+TSAP	0.249[14]	9.00[14]				244[15] 180[16]		

a) Taken from the X-ray crystallographic structure of GdTCE-DOTA, which maintains a SAP conformation. b) Taken from the X-ray crystallographic structure of GdDOTMA, which maintains a TSAP conformation. C) Taken from the S-RRRR-LnNO2 BnDOTMA complex, which is a constrained conformer in SAP geometry. d) Taken from the S-SSSS-LnNO2 BnDOTMA which is a contrained conformer in TSAP geometry. e) Taken from $[Eu(DOTAM)(H2O)]^{3+}$. f) taken from $[Eu(dtma)(H2O)]^{3+}$

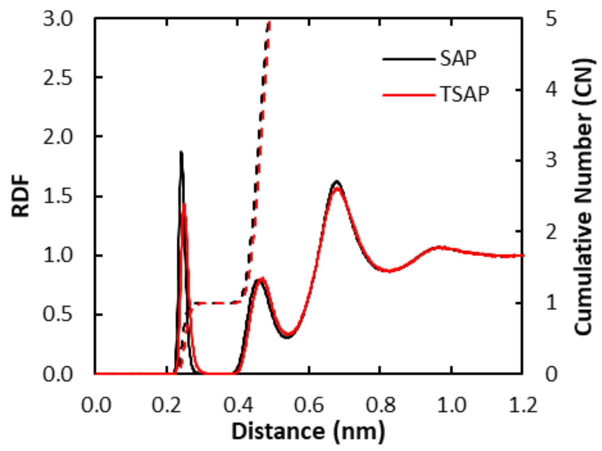

Figure S14. Gd^{3+}-O_{water} RDFs (lines) and cumulative number RDFs (dotted lines) for the topology of $[Gd(DOTA)]^-$ in the SAP (black) and TSAP (red).

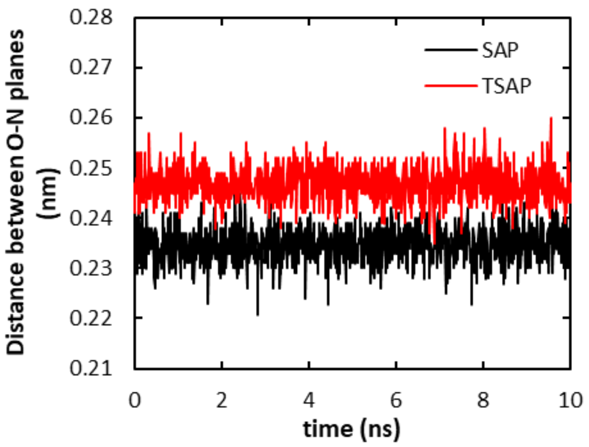

Figure S15. Distance between the plane formed by the coordinated oxygens and the plane formed by the nitrogen atoms of the $[Gd(DOTA)]^-$ in the SAP conformations (black line) and the TSAP conformation (red line) during the MD simulation.

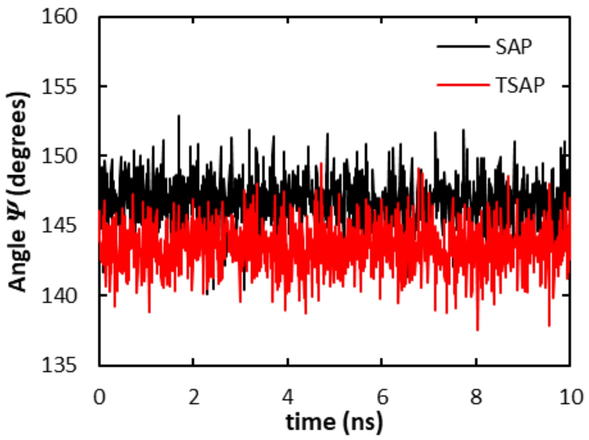

Figure S16. Opening angle Ψ of the $[Gd(DOTA)]^-$ in the SAP conformations (black line) and the TSAP conformation (red line) during the MD simulation.

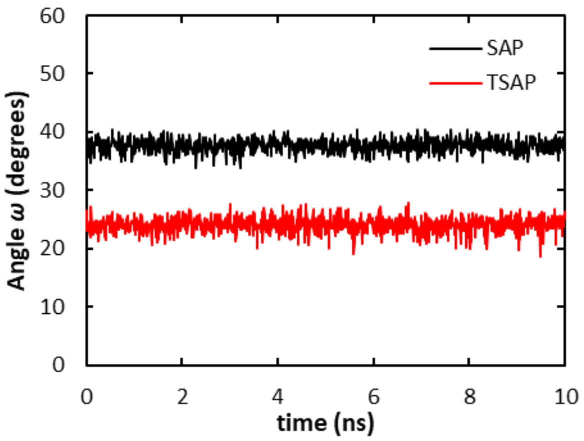

Figure S17. Twist angle ω of the $[Gd(DOTA)]^-$ in the SAP conformation (black line) and the TSAP conformation (red line) during the MD simulation.

References

(1) Li, P.; Merz, K. M. MCPB.Py: A Python Based Metal Center Parameter Builder. *J. Chem. Inf. Model.* **2016**, *56* (4), 599–604. https://doi.org/10.1021/acs.jcim.5b00674.

(2) M.J. Abraham, D. van der Spoel, E. Lindahl, B. Hess, and the GROMACS development Team. GROMACS User Manual Version 2019.3; 2019. https://doi.org/10.5281/zenodo.3243834.

(3) Henriques, E. S.; Bastos, M.; Geraldes, C. F. G. C.; João Ramos, M. Computational Approaches to the Study of Some Lanthanide (III)-Polyazamacrocyclic Chelates for Magnetic Resonance Imaging. *Int. J. Quantum Chem.* **1999**, *73* (2), 237–248. https://doi.org/10.1002/(SICI)1097-461X(1999)73:2<237::AID-QUA17>3.0.CO;2-W.

(4) Henriques, E. S.; Geraldes, C. F. G. C.; Ramos, M. J. Modelling Studies in Aqueous Solution of Lanthanide (III) Chelates Designed for Nuclear Magnetic Resonance Biomedical Applications. *Mol. Phys.* **2003**, *101* (15), 2319–2333. https://doi.org/10.1080/0026897031000108023.

(5) Dimelow, R. J.; Burton, N. A.; Hillier, I. H. The Dynamics of Water Exchange in Gadolinium DOTA Complexes Studied by Transition Path Sampling and Potential of Mean Force Methods. *Phys. Chem. Chem. Phys.* **2007**, *9* (11), 1318. https://doi.org/10.1039/b617068d.

(6) Dolg, M.; Stoll, H.; Savin, A.; Preuss, H. Energy-Adjusted Pseudopotentials for the Rare Earth Elements. *Theor. Chim. Acta* **1989**, *75* (3), 173–194. https://doi.org/10.1007/BF00528565.

(7) Dolg, M.; Stoll, H.; Preuss, H. A Combination of Quasirelativistic Pseudopotential and Ligand Field Calculations for Lanthanoid Compounds. *Theor. Chim. Acta* **1993**, *85* (6), 441–450. https://doi.org/10.1007/BF01112983.

(8) Li, P.; Song, L. F.; Merz, K. M. Parameterization of Highly Charged Metal Ions Using the 12-6-4 LJ-Type Nonbonded Model in Explicit Water. *J. Phys. Chem. B* **2015**, *119* (3), 883–895. https://doi.org/10.1021/jp505875v.

(9) Peters, J. A.; Djanashvili, K.; Geraldes, C. F. G. C.; Platas-Iglesias, C. The Chemical Consequences of the Gradual Decrease of the Ionic Radius along the Ln-Series. *Coord. Chem. Rev.* **2020**, *406*, 213146. https://doi.org/10.1016/j.ccr.2019.213146.

(10) Aime, S.; Botta, M.; Garda, Z.; Kucera, B. E.; Tircso, G.; Young, V. G.; Woods, M. Properties, Solution State Behavior, and Crystal Structures of Chelates of DOTMA. *Inorg. Chem.* **2011**, *50* (17), 7955–7965. https://doi.org/10.1021/ic2012827.

(11) Woods, M.; Aime, S.; Botta, M.; Howard, J. A. K.; Moloney, J. M.; Navet, M.; Parker, D.; Port, M.; Rousseaux, O. Correlation of Water Exchange Rate with Isomeric Composition in Diastereoisomeric Gadolinium Complexes of Tetra(Carboxyethyl)Dota and Related Macrocyclic Ligands. *J. Am. Chem. Soc.* **2000**, *122* (40), 9781–9792. https://doi.org/10.1021/ja994492v.

(12) Woods, M.; Botta, M.; Avedano, S.; Wang, J.; Sherry, A. D. Towards the Rational Design of MRI Contrast Agents: A Practical Approach to the Synthesis of Gadolinium Complexes That Exhibit Optimal Water Exchange. *Dalt. Trans.* **2005**, No. 24, 3829. https://doi.org/10.1039/b510778d.

(13) Dunand, F. A.; Aime, S.; Merbach, A. E. First 17O NMR Observation of Coordinated Water on Both Isomers of [Eu(DOTAM)(H2O)]3+: A Direct Access to Water Exchange and Its Role in the Isomerization. *J. Am. Chem. Soc.* **2000**, *122* (7), 1506–1512. https://doi.org/10.1021/ja993204s.

(14) Moreau, J.; Guillon, E.; Pierrard, J.-C.; Rimbault, J.; Port, M.; Aplincourt, M. Complexing Mechanism of the Lanthanide Cations Eu3+, Gd3+, and Tb3+ with 1,4,7,10-Tetrakis(Carboxymethyl)-1,4,7,10-Tetraazacyclododecane (Dota)—Characterization of Three Successive Complexing Phases: Study of the Thermodynamic and Structural Properties Of. *Chem. - A Eur. J.* **2004**, *10* (20), 5218–5232. https://doi.org/10.1002/chem.200400006.

(15) Powell, D. H.; Dhubhghaill, O. M. N.; Pubanz, D.; Helm, L.; Lebedev, Y. S.; Schlaepfer, W.; Merbach, A. E. Structural and Dynamic Parameters Obtained from 17O NMR, EPR, and NMRD Studies of Monomeric and Dimeric Gd3+ Complexes of Interest in Magnetic Resonance Imaging: An Integrated and Theoretically Self-Consistent Approach. *J. Am. Chem. Soc.* **1996**, *118* (39), 9333–9346. https://doi.org/10.1021/ja961743g.

(16) Dunand, F. A.; Borel, A.; Helm, L. Gd(III) Based MRI Contrast Agents: Improved Physical Meaning in a Combined Analysis of EPR and NMR Data? *Inorg. Chem. Commun.* **2002**, *5* (10), 811–815. https://doi.org/10.1016/S1387-7003(02)00575-0.

(17) Dunand, F. A.; Dickins, R. S.; Parker, D.; Merbach, A. E. Towards Rational Design of Fast Water-Exchanging Gd(Dota-Like) Contrast Agents? Importance of the M/m Ratio. *Chem. - A Eur. J.* **2001**, *7* (23), 5160–5167. https://doi.org/10.1002/1521-3765(20011203)7:23<5160::AID-CHEM5160>3.0.CO;2-2.
